# Beyond Boltzmann–Gibbs–Shannon in Physics and Elsewhere

**DOI:** 10.3390/e21070696

**Published:** 2019-07-15

**Authors:** Constantino Tsallis

**Affiliations:** 1 Centro Brasileiro de Pesquisas Físicas and National Institute of Science and Technology for Complex Systems–Rua Dr. Xavier Sigaud 150, Rio de Janeiro 22290-180, Brazil; tsallis@cbpf.br; 2 Santa Fe Institute–1399 Hyde Park Road, Santa Fe, NM 87501, USA; 3 Complexity Science Hub Vienna–Josefstädter Strasse 39, 1080 Vienna, Austria

**Keywords:** complex systems, nonadditive entropies, nonextensive statistical mechanics, beyond Boltzmann–Gibbs–Shannon

## Abstract

The pillars of contemporary theoretical physics are classical mechanics, Maxwell electromagnetism, relativity, quantum mechanics, and Boltzmann–Gibbs (BG) statistical mechanics –including its connection with thermodynamics. The BG theory describes amazingly well the thermal equilibrium of a plethora of so-called simple systems. However, BG statistical mechanics and its basic additive entropy $S_{BG}$ started, in recent decades, to exhibit failures or inadequacies in an increasing number of complex systems. The emergence of such intriguing features became apparent in quantum systems as well, such as black holes and other area-law-like scenarios for the von Neumann entropy. In a different arena, the efficiency of the Shannon entropy—as the BG functional is currently called in engineering and communication theory—started to be perceived as not necessarily optimal in the processing of images (e.g., medical ones) and time series (e.g., economic ones). Such is the case in the presence of generic long-range space correlations, long memory, sub-exponential sensitivity to the initial conditions (hence vanishing largest Lyapunov exponents), and similar features. Finally, we witnessed, during the last two decades, an explosion of asymptotically scale-free complex networks. This wide range of important systems eventually gave support, since 1988, to the generalization of the BG theory. Nonadditive entropies generalizing the BG one and their consequences have been introduced and intensively studied worldwide. The present review focuses on these concepts and their predictions, verifications, and applications in physics and elsewhere. Some selected examples (in quantum information, high- and low-energy physics, low-dimensional nonlinear dynamical systems, earthquakes, turbulence, long-range interacting systems, and scale-free networks) illustrate successful applications. The grounding thermodynamical framework is briefly described as well.

## 1. Introduction

Relativity generalizes Newtonian mechanics in order to also include velocities close to that of light; along a different line, quantum mechanics also generalizes Newtonian mechanics in order to also include small masses. Since the magnificent nineteenth-century contributions by Boltzmann and by Gibbs (BG) and later on by Bose and Einstein, as well as by Fermi and Dirac for quantum systems, the BG theory satisfactorily addresses an impressive number of problems. And still, it comes up short for many others, frequently referred to as complex systems. To be more precise, their collective stationary states do not always correspond to what is usually referred to as thermal equilibrium.

It can be argued that some sort of scenario has been emerging along the last three or four decades, calling for a paradigm shift in what concerns the historical axioms leading to the BG entropy and to its associated statistical mechanics [1,2,3]. The crucial point turned out to be, as detailed hereafter, the additivity of the BG form. The simplest form of the BG entropic functional is

(1) $$S_{BG}=-k\sum_{i=1}^{W}p_{i}\ln p_{i}\;\;\;\left(\sum_{i=1}^{W}p_{i}=1\right)\;,$$

where *W* is the total number of (microscopic) possibilities, and *k* is a conventional positive constant (physicists identify *k* with the Boltzmann constant $k_{B}$; information theoreticians use k=1). This form leads, in the continuum limit, to the historical expression $S_{BG}=-k\int dx\;p\left(x\right)\ln p\left(x\right)$ (with ∫dxp(x)=1, where x is a continuous multidimensional random variable) and to what is known as the von Neumann entropy $S_{BG}-kTr\rho \ln\rho$ [4] for quantum systems, ρ being the density operator. The expression (1) is referred to as Shannon entropy in communication theory [5]. If we have a system composed of two probabilistically independent subsystems *A* and *B* (i.e., $p_{ij}^{A+B}=p_{i}^{A}p_{j}^{B}$), we straightforwardly verify that $S_{BG}$ satisfies the Penrose requirement [6] for entropic additivity, namely S(A+B)=S(A)+S(B).

Within an information theoretical framework not necessarily related to physics, in 1961 Rényi introduced [7] the most general additive form, which contains $S_{BG}$ as a particular case, namely

(2) $$S_{q}^{R}=k\frac{\ln\sum_{i=1}^{W}p_{i}^{q}}{1-q}\;\;\;\left(q\in R;S_{1}^{R}=S_{BG}\right)\;.$$

Within that information theory scenario, various other possible generalizations of $S_{BG}$ emerged, e.g., in 1967 by Havrda and Charvat [8], in 1975 by Sharma and Mittal [9]. Independently from such predecessors as mathematical possibilities and inspired by multifractals, in 1988 [10] the generalization of BG statistical mechanics itself through the optimization of the entropy $S_{q}$ was proposed in physics, defined as

(3) $$S_{q}=k\frac{1-\sum_{i=1}^{W}p_{i}^{q}}{q-1}\;\;\;\left(q\in R;S_{1}=S_{BG}\right)\;.$$

This entropy satisfies, for independent *A* and *B*, the following nonadditive property:

(4) $$\frac{S_{q}\left(A+B\right)}{k}=\frac{S_{q}\left(A\right)}{k}+\frac{S_{q}\left(B\right)}{k}+\left(1-q\right)\frac{S_{q}\left(A\right)}{k}\frac{S_{q}\left(B\right)}{k}\;,$$

hence

(5) $$S_{q}\left(A+B\right)=S_{q}\left(A\right)+S_{q}\left(B\right)+\frac{1-q}{k}S_{q}\left(A\right)\;S_{q}\left(B\right)\;,$$

which recovers the BG additivity in the (1−q)/k→0 limit. The unprecedented use of a nonadditive entropy (a conceptual possibility which, in one way or another, had already been considered historically [11,12,13]) in order to generalize the BG statistical mechanics, opened a door that has been being widely explored since 1988 [14,15]. It is for a wide class of anomalous situations, including analogous geometrical random systems such as asymptotically scale-free networks (see, for instance, [16,17,18] and references therein involving distance-dependent couplings of the type $r^{-\alpha_{a}}$; for the particular case of $\alpha_{A}=0$, see [19,20,21,22]) that non-Boltzmannian entropies and related formalisms become useful. A neat explanation of the difference between entropic additivity and entropic extensivity will be provided below. This is a most important issue, since these two definitively distinct properties are very frequently confused by physicists and others.

Other extensions of the BG entropy and its associated statistical mechanics followed in physics and elsewhere, for example, in 1998 by Borges and Roditi [23] and its particular instance in 2001 by Kaniadakis [24,25,26,27], in 1997 by Abe [28], in 1998 by Landsberg and Vedral [29], in 1999 by Curado [30,31], in 1999 by Anteneodo and Plastino [32], in 2005 by Tsekouras and Tsallis [33], in 2003 by Tsallis and Souza 2003 [34] (within the context of Beck–Cohen superstatistics [35]), in 2007 by Schwammle andTsallis [36], the δ-entropy in 2007 and 2009 by Shafee, Tsallis, and Ubriaco [37,38,39], generalized in 2013 by Tsallis and Cirto [40], in 2011 by Hanel and Thurner [41], in 2011 by Tempesta [42] (including its intriguing connection with the Riemann zeta function; see also [43]), in 2016 by Curado, Tempesta, and Tsallis [44], in 2016 by Tempesta [45] (see also [43]), in 2018 by Jensen, Pazuki, Pruessner, and Tempesta [46], among various other developments. These entropic functionals have many connections and predecessors in areas such as cybernetics, information theory, engineering, communication theory, ecology, and information geometry. All of them recover the celebrated entropy $S_{BG}$ as a particular case, with the unique exceptions of the exponential-form ones, namely [30,33]. Only two of all these entropic functionals are additive, namely the Boltzmann–Gibbs–von Neumann–Shannon $S_{BG}$ and the Rényi [7] ones. All the others are generically nonadditive.

As is well known, the entropy $S_{BG}$ and its associated statistical mechanics enable the correct calculation of a large variety of thermostatistical properties at or near the thermal equilibrium of uncountable so-called simple systems. But when it comes to wide classes of so-called complex systems, the BG theory fails. Due to this fact, many attempts have emerged using either the Rényi entropy or some of the nonadditive ones, most frequently $S_{q}$, for a variety of applications in natural, artificial, and social systems. In what follows, we focus on some selected illustrations, based on the volume of applications and connections that are exhibited in the constantly growing literature.

## 2. Non-Boltzmannian Entropy Measures and Distributions

### 2.1. Rényi Entropy

As already mentioned, Rényi entropy was long ago introduced [7] in information theory as the most general additive entropic functional that recovers the BG one as a particular case. It satisfies the first three axioms of Shannon [5] (basically that entropy depends smoothly and only on probabilities, that it is maximal for equal probabilities, and that it is additive, meaning that the entropy of a system composed of independent subsystems is simply the sum of the entropies), but violates the (celebrated) fourth one, namely the one that refers to the grouping property. It was initially used in the context of strongly chaotic nonlinear dynamical systems, this is to say systems whose dynamics are exponentially sensitive in time to the initial conditions, i.e., positive Lyapunov exponents, mathematically speaking (see, for instance, [47]). But in recent years, the Rényi entropy has become popular in quantum information, and more precisely, to characterize quantum entanglement. This specific use is pedagogically illustrated in [48] for a system of *N* identical fermions. If we denote by ρ the density matrix of a pure or mixed state of *N* fermions and by $\rho_{r}$ the density matrix of the single-particle reduced state, it can be proved for any q≥1, that $S_{q}^{R}\left[\rho_{r}\right]/k>S_{q}^{R}\left[\rho \right]/k+\ln N$ implies that ρ corresponds to a necessarily quantum entangled state, i.e., the state is not separable [48]. This inequality can therefore be used as a criterion for identifying quantum entanglement. The criterion becomes stronger as *q* increases from one to infinity, in the sense that the larger the value of *q*, the larger the number of entangled states that are detected. For some particular situations, the q→∞ limit corresponds, in fact, to the necessary and sufficient conditions for the system to be entangled or separable (see, for instance, [49], by recalling that $S_{q}^{R}$ monotonically depends on $S_{q}$).

### 2.2. q-Entropy and q-Exponential Distribution

Let us focus now on the profusely used entropy $S_{q}$. As mentioned above, it was introduced in 1988 [10] with the purpose of generalizing BG statistical mechanics itself. This entropic functional is simply related to the Rényi functional through a monotonic function. There are, however, three crucial differences among them. First, $S_{q}^{R}$ is additive for all values of *q*, whereas $S_{q}$ is nonadditive for all q≠1. Second, the Rényi entropy has a definite concavity only for q≤1 (concave for 0<q≤1 and convex for q<0; q=0 corresponds to an anomalous frontier), whereas the *q*-entropy has a definite concavity for all values of *q* (concave for q>0 and convex for q<0); this important difference comes from the fact that concavity is not preserved through the already mentioned monotonicity between the Rényi entropy and $S_{q}$ (concavity is an important mathematical property that is directly related to thermodynamical stability, or in other words, to the fact that the specific heat of the BG canonical ensemble of any system must be non-negative). Third, the extremal value of the Rényi entropy (at equiprobability) recovers Boltzmann’s celebrated expression, namely $S_{q}^{R}=k\ln W$, for all values of *q*, whereas the (*q*-dependent) extremal value of the *q*-entropy is given by $S_{q}=k\ln_{q}W$, where the *q*-logarithmic function is defined as $\ln_{q}z\equiv \frac{z^{1-q}-1}{1-q}$, with $\ln_{1}z=\ln z$. Further details are discussed in [50].

The entropy $S_{q}$ can be conveniently rewritten as follows:

(6) $$S_{q}=k\sum_{i=1}^{W}p_{i}\ln_{q}\frac{1}{p_{i}}=-k\sum_{i=1}^{W}p_{i}^{q}\ln_{q}p_{i}=-k\sum_{i=1}^{W}p_{i}\ln_{2-q}p_{i}\;.$$

Its optimization in the presence of appropriate norm and energy constraints yields the following probability distribution:

(7) $$p_{i}=\frac{e_{q}^{-\beta_{q}E_{i}}}{\sum_{j=1}^{W}e_{q}^{-\beta_{q}E_{j}}},$$

where $\left\{E_{i}\right\}$ are the total energy eigenvalues, the *q*-exponential function $e_{q}^{z}\equiv {\left[1+\left(1-q\right)z\right]}^{\frac{1}{1-q}}$ is defined as the inverse of $\ln_{q}z$, and $\beta_{q}\equiv 1/kT_{q}>0$ is related to the constraints that have been imposed (see details in [10,51,52]); the following properties are satisfied: $e_{q}^{z}e_{2-q}^{-z}={\left(e_{q}^{z}\right)}^{q}e_{1/q}^{-qz}=1$, $e_{q}^{x+y+(1-q)xy}=e_{q}^{x}e_{q}^{y}$, and $e_{q}^{z}∼1+z\;\left(z\to 0\right)$, for all values of *q*. This is how the central operational ingredient of the BG theory, namely the BG exponential weight [1,2,3], is herein generalized. At this point, it is worth mentioning a general property, satisfied by entropic functionals. To illustrate this property, we can focus on, say, $S_{q}$. Any monotonic function of $S_{q}$ (e.g., Rényi entropy) is optimized by one and the same distribution if the constraints are precisely the same. This by no means implies that the entire associated statistical mechanics and thermodynamics are also one and the same. An obvious illustration of this fact is that both $-k\sum_{i=1}^{W}p_{i}\ln p_{i}$ and $-k{\left[\sum_{i=1}^{W}p_{i}\ln p_{i}\right]}^{3}$ are optimized, under the same constraints, by the celebrated BG factor. However, the corresponding statistical mechanics are completely different. Indeed, the microscopical interpretation of the thermodynamical entropy as being $S_{BG}$ satisfies, as is well known, a plethora of correct standard thermodynamical relations, whereas the use of its cube instead would violate plenty of them.

Within many applications of *q*-statistics [15], let us present here four selected case studies, two in high-energy physics (on Earth and in outer space),and the other two in low-energy physics (granular matter and cold atoms).

**Figure 1:**
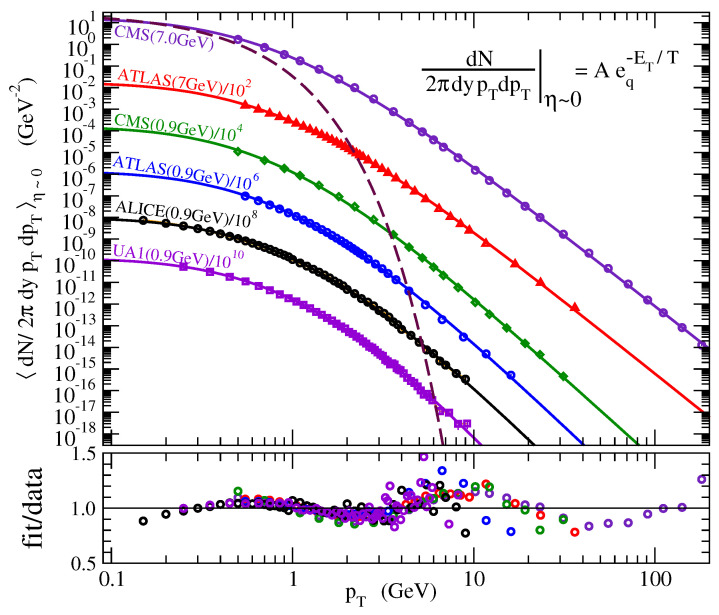

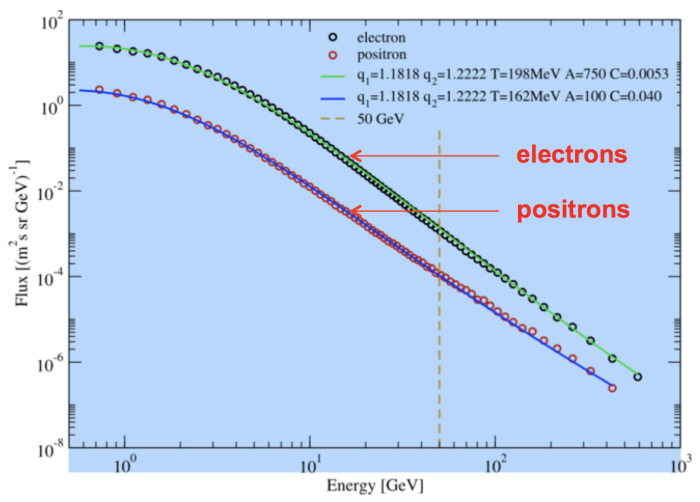
**Applications in high-energy physics**. **Top:** Comparison of $Ae_{q}^{-E_{T}/T}$, where $E_{T}=\sqrt{m^{2}+p_{T}^{2}}$, with the experimental transverse momentum distribution of hadrons in pp collisions at central rapidity *y*. The corresponding Boltzmann–Gibbs (purely exponential) distribution is illustrated as a dashed curve. For a better visualization, both the data and the analytical curves have been divided by a constant factor, as indicated. As shown, the fittings are amazingly good over as many as 14 ordinate decades! Such a situation appears to be unprecedented. Indeed, so many experimental decades within a single experiment is a rather unique circumstance, which exhibits the talent of the experimental effort involved. To realistically appreciate this, such curves can be compared to, say, those exhibiting the crossover from Newtonian to Einstein mechanics at increasingly large values of the momentum. Indeed, if we consider the case of, say, protons within cosmic rays up to the Extreme Energy Cosmic Ray detection on Earth, we have 11 ordinate decades between the departure of the Einstein relation $E=\sqrt{m^{2}c^{4}+p^{2}c^{2}}$ from the classical relation $E=mc^{2}+p^{2}/2m$ up to the relativistic upper experimental limit. The ratios data/fit are shown at the bottom, where a roughly log-periodic behavior is observed on top of the *q*-exponential one. Such log-periodic curves have been remarkably well fitted by introducing in the *q*-index a small imaginary part (e.g., q=1.14+i0.03) [61,62]. From [56]. **Bottom:** The measured AMS-02 data are very well fitted by linear combinations of escort and standard distributions with $q_{1}=13/11=1.1818\ldots$ and $q_{2}=1/\left(2-q_{1}\right)=11/9=1.2222\ldots$ From [63].

Let us start with high-energy physics. Rolf Hagedorn and others suggested in the 1960s that the distributions of hadronic transverse momenta emerging after high-energy collisions could be explained in terms of BG statistics. This interesting idea was quite successful for collisions of not-too-large transverse momenta, but started failing when larger and larger momenta gradually started becoming prominent, and more precisely when modern accelerators enabled collisions at higher and higher center-of-mass energies. However, in 2000, a successful description was advanced for electron–positron collisions by replacing, within Hagedorn theory, the BG factor by the *q*-exponential one [53]. After this pioneering attempt, many other successful calculations were performed, focusing on experiments done at RHIC/Brookhaven (STAR, PHENIX Collaborations) and LHC/CERN (ALICE, CMS, ATLAS, LHCb Collaborations) [54,55,56,57,58,59,60,61,62], as well as on observations in outer space [63]. For illustrations in these areas of research, see Figure 1. The state-of-the-art is that *q* monotonically depends on the collision center-of-mass energy, being q≃1 for low-energy collisions and approaching q≃1.2 for high-energy collisions, of the order of the extreme-energy cosmic rays (close to $10^{20}$ eV). The original Hagedorn theory is thus recovered naturally at the q→1 limit. As another trivial consequence of the definition of the *q*-exponential function, the forms of the distributions asymptotically coincide in the low-momenta region for all center-of-mass energies, which constitutes another limit at which the Hagedorn theory is recovered.

The other high-energy application concerns the observation of matter (electrons) and antimatter (positrons) in outer space with the Alpha Magnetic Spectrometer (AMS-02), led by Samuel Ting and shown in Figure 1. The measured energy-dependent fluxes have been recently shown, by Yalcin and Beck [63], to be well fitted by linear combinations of standard and escort *q*-exponentials whose two *q* values, namely $q_{1}$ and $q_{2}$, are related through a simple combination of the so-called additive duality (q→2−q) and multiplicative duality (q→1/q), discussed in [64]; the present specific values of $q_{1}$ and $q_{2}$ are established on the basis of the involved number of degrees of freedom.

**Figure 2:**
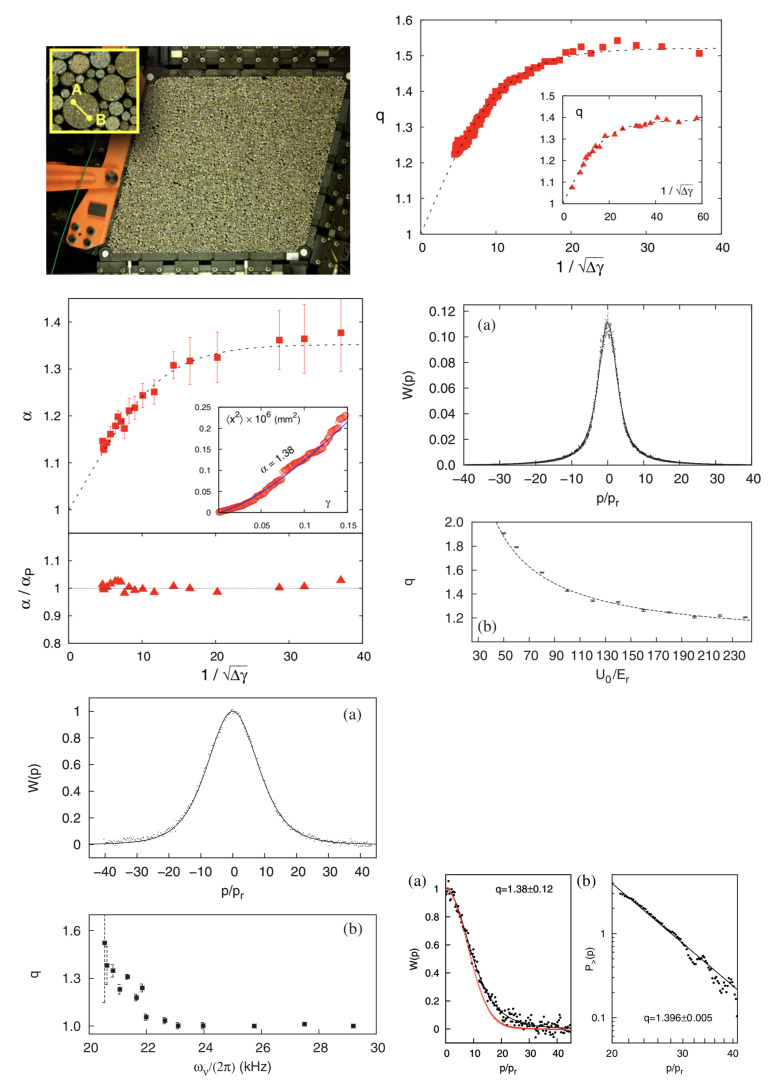
**Applications in low-energy physics**. Experimental verification in granular matter of the scaling relation predicted in 1996 [67]. (**Top left:**) Type of apparatus that is used (from [71]); (**top right:**) dependence of the index *q* of the *q*-Gaussian distribution of fluctuations on a wide range of the experimental parameter $1/\sqrt{\Delta \gamma }$; (**middle left:**) dependence of the anomalous diffusion exponent α ($x^{2}$ scales with $t^{\alpha }$) on the same experimental parameter and verification, within a 2% error bar, of the 1996 prediction $\alpha =\alpha_{P}\equiv 2/\left(3-q\right)$ [67]. Notice that in the $1/\sqrt{\Delta \gamma }\to 0$ limit, the BG values (q,α)=(1,1) emerge as the expected extrapolations. From [70]. Experimental verification for cold atoms of the 2003 prediction by Lutz [72]. (**Middle right:**) (**a, in Middle right**) results of quantum Monte Carlo simulations for the momentum distribution of atoms cooled in a 1D optical lattice. The data points correspond to the average of $10^{4}$ atomic trajectories. For each trajectory, the atom is initially in the ground state of a given well. The depth of the optical lattice is $U_{0}=60E_{r}$. The line is the best fit to the data with a *q*-Gaussian with q=1.791±0.004 (adjusted $R^{2}=0.995$). (**b, in Middle right**) Values of *q* as a function of the depth of the optical potential. The data points correspond to the full quantum Monte Carlo simulations, the line representing the analytical prediction $q=1+44E_{r}/U_{0}$ [72]. (**Bottom left:**) (**a, in Bottom left**) experimental results for the momentum distribution of cold atoms in a 3D dissipative optical lattice (data points) and their best fit *q*-Gaussian (solid line). The obtained *q* value (=1.310±0.015) is derived by fitting only the right part of the momentum distribution (adjusted $R^{2}=0.9985$). The parameter of the optical lattice is $\omega_{v}/\left(2\pi \right)=20.8\;\mathrm{kHz}$. The distribution is normalized so that its maximum equals unity. (**b, in Bottom left**) Values for *q* as a function of the vibrational frequency at the bottom of the well, as obtained by fitting the experimental data with a *q*-Gaussian. (**Bottom right:**) (**a, in Bottom right**) experimental results for the atomic momentum distribution (black data points) and their best fit with a *q*-Gaussian (black solid line). The value of *q* is indicated in the figure (adjusted $R^{2}=0.9985$). The parameter of the optical lattice is $\omega_{v}/\left(2\pi \right)=27.5\;\mathrm{kHz}$. For comparison, a Gaussian is indicated as well (red line). (**b, in Bottom right**) The data points for the distribution in the high-momenta region. The solid line represents the best power-law fit. From [73,74].

Turning to low-energy applications, let us focus now on recent granular matter experiments. In 1995, it was shown by Plastino and Plastino [65] that the so-called porous medium equation in the presence of a confined potential is connected to the $S_{q}$ entropy and its *q*-Gaussian extremizing distribution (proportional to $e_{q}^{-\beta_{q}x^{2}}$). The connection is, in fact, a fundamental one, namely that the stationary state of the nonlinear Fokker–Planck equation precisely coincides with the distribution that extremizes the entropy $S_{q}$ under the corresponding constraints, and also satisfies an H-theorem, for all values of *q* [66]. On this basis, a novel scaling relation was thereafter established [67], namely α=2/(3−q), where the anomalous diffusion exponent α is defined through the fact that the square displacement $x^{2}$ scales like $t^{\alpha }$ (notice that q=1 yields the classical Brownian motion exponent α=1). Preliminary confirmations emerged in an experimental study of the motion of *Hydra* cells [68] and in a computational approach of the XY model with long-range interactions [69]. But the experimental validation of the scaling relation on a wide physical range, on granular matter in fact [70], only arrived 20 years later (see Figure 2).

The other low-energy application concerns the motion of cold atoms in optical dissipative lattices. It was suggested by Lutz in 2003 [72] that under appropriate conditions (involving a damping mechanism, e.g., the so-called Sisyphus cooling), the distribution of velocities of atoms should be not Maxwellian but rather *q*-Gaussian with $q=1+44\frac{E_{r}}{U_{0}}$, where $E_{r}$ is the atomic recoil energy and $U_{0}$ is the amplitude of the periodic potential produced by the laser field. Three years later, in 2006, his prediction was computationally and experimentally verified [73]. This impressive confirmation was computationally performed both through quantum Monte Carlo and experimentally with Cs atoms; see Figure 2. The entire idea was further discussed in [74].

Let us now reproduce here some strongly suggestive numerical results concerning low-dimensional maps, namely the d=1 logistic map (dissipative) and the d=2 standard map (conservative).

The logistic map can be defined as follows:

(8) $$x_{t+1}=1-ax_{t}^{2}\;\;\;\left(t=0,1,2,3\ldots ;\;x_{t}\in \left[-1,1\right];\;a\in \left[0,2\right]\right).$$

**Figure 3:**
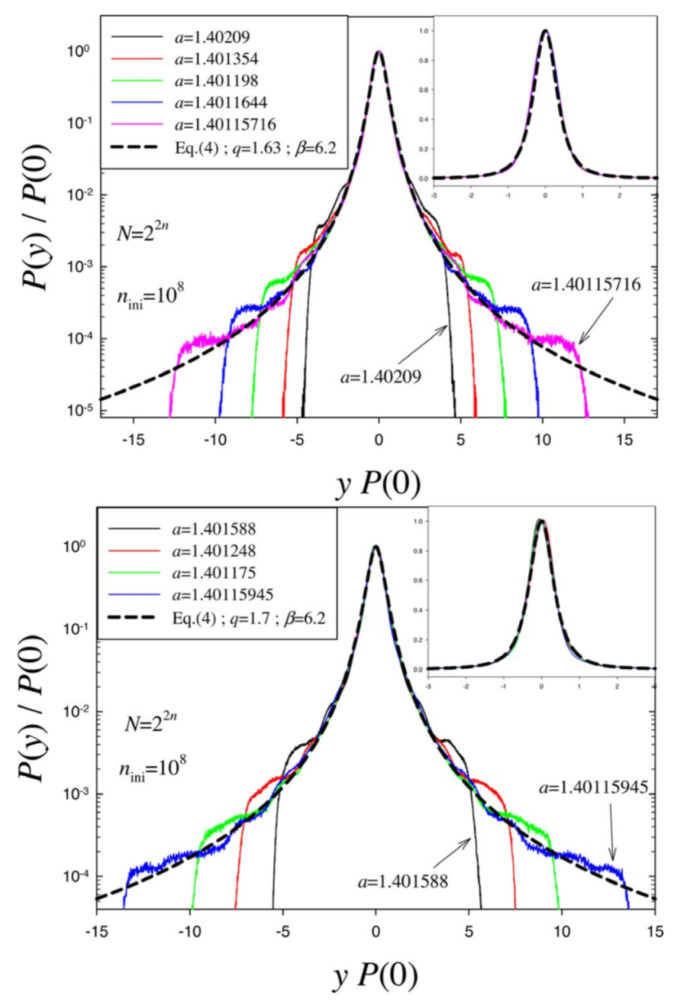
Data collapse of probability density functions for the cases $N=2^{2n}$, where 2n is odd (**top**), or even (**bottom**). As *n* increases, a good fit using a *q*-Gaussian $P\left(y\right)/P\left(0\right)=e_{q}^{-\beta {\left[yP\left(0\right)\right]}^{2}}$ with (q,β)≃(1.68,6.2) (**top**) and (q,β)≃(1.70,6.2) (**bottom**) is obtained for increasingly large regions. *Inset:* Linear-linear plots of the data for a better visualization of the central part. From [75].

For $a<a_{c}=1.401155189092\ldots$, the (unique) Lyapunov exponent λ is non positive; for $a=a_{c}$, frequently referred to in the literature as the Feigenbaum point, the Lyapunov exponent vanishes; and for $a>a_{c}$, λ can be negative, zero, or positive, being λ=ln2 for a=2, the most chaotic case for this paradigmatic map. An interesting quantity to focus on is the attractor of the sum of many successive iterations of $x_{t}$. For a=2, the attractor is a Gaussian, as expected from the central limit theorem. For $a=a_{c}$ instead, the attractor approaches *q*-Gaussians, as shown in Figure 3 ([75]).

Various other q≠1 relevant quantities, including the *q*-generalization of the Pesin identity, are also available at the Feigenbaum point [76,77,78,79].

The standard map is defined as follows:

(9) $$\begin{array}{ccc} p_{t+1} & = & p_{t}-K\;sinx_{t} \end{array}$$

(10) $$\begin{array}{ccc} x_{t+1} & = & x_{t}+p_{t+1}\;\;\;\left(t=0,1,2,3\ldots ;\;K\geq 0\right)\;, \end{array}$$

**Figure 4:**
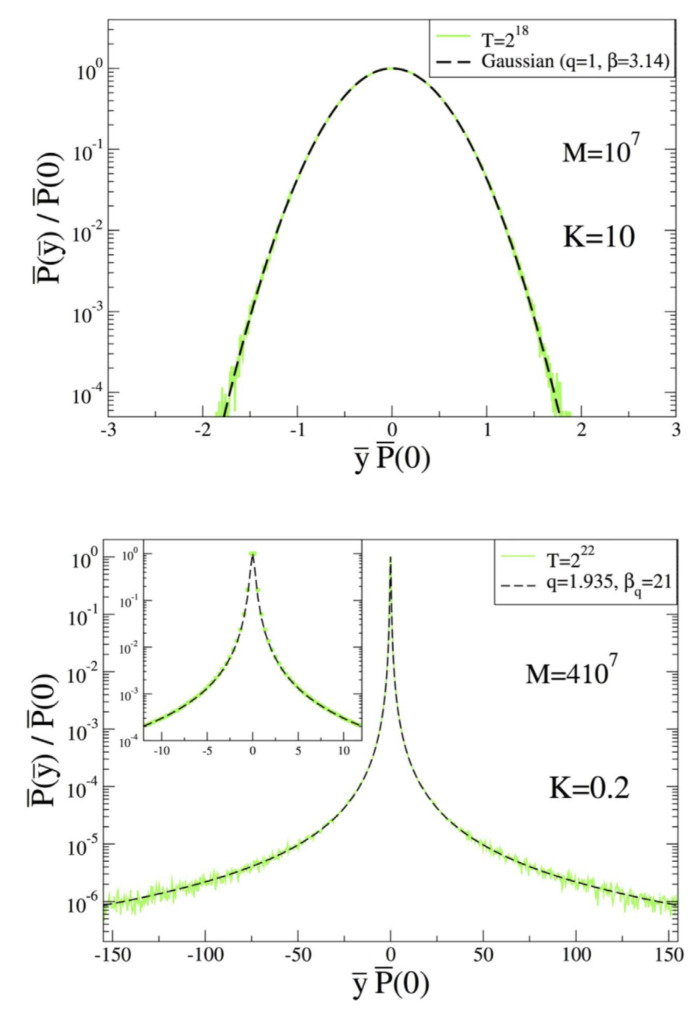
Data. Normalized probability distribution function of the attractors, where *T* is the number of terms in the sum and *M* is the number of initial conditions. (**Top:**) For K=10 with $T=2^{18}$. (**Bottom:**) For K=0.2 with $T=2^{22}$. (In the Inset, the central part is zoomed for a better visualization). From [80].

$x_{t}$ and $y_{t}$ being defined as real numbers modulo 2π. Being conservative, this well known map has two Lyapunov exponents opposed by their sign, i.e., λ and −λ with λ≥0. When *K* vanishes, λ vanishes in the full phase-space; when *K* is very large, λ is positive in the full phase-space. In an interesting paper, Tirnakli and Borges [80] have recently focused on the sum of a large number of successive iterations of $x_{t}$. Their main numerical results concerning the attractors that emerge are shown in Figure 4. For K=10, the Lyapunov exponent is neatly positive, and the attractor is a Gaussian, in agreement with the central limit theorem. For K=0.2 (and even for K=0, not shown here) the Lyapunov exponent is very close to zero (or just zero) and the attractor appears to be a *q*-Gaussian with (q,β)≃(1.935,21) along a considerable number of decades.

### 2.3. Kaniadakis Entropy and κ-Exponential Distribution

In 2001 [24,25,26,27], Kaniadakis introduced the following nonadditive entropy:

(11) $$S_{\kappa }=k\frac{\frac{1}{1-\kappa }\sum_{i=1}^{W}p_{i}^{1-\kappa }-\frac{1}{1+\kappa }\sum_{i=1}^{W}p_{i}^{1+\kappa }}{2\kappa }\;\;\;\left(\kappa \in R;\;S_{0}=S_{BG}\right)\;.$$

It is directly related to a linear combination of two *q*-entropies involving q=1+κ and constitutes a particular instance of the Borges–Roditi entropy [23]. The Kaniadakis entropy was defined in order to satisfy the simple symmetry $S_{\kappa }=S_{-\kappa }$ and is, under appropriate constraints, extremized in terms of the κ-exponential function defined as

(12) $$e_{\kappa }^{z}={\left[\sqrt{1+\kappa^{2}z^{2}}+\kappa z\right]}^{\frac{1}{\kappa }}\;\;\;\left(e_{0}^{z}=e^{z}\right)\;.$$

**Figure 5:**
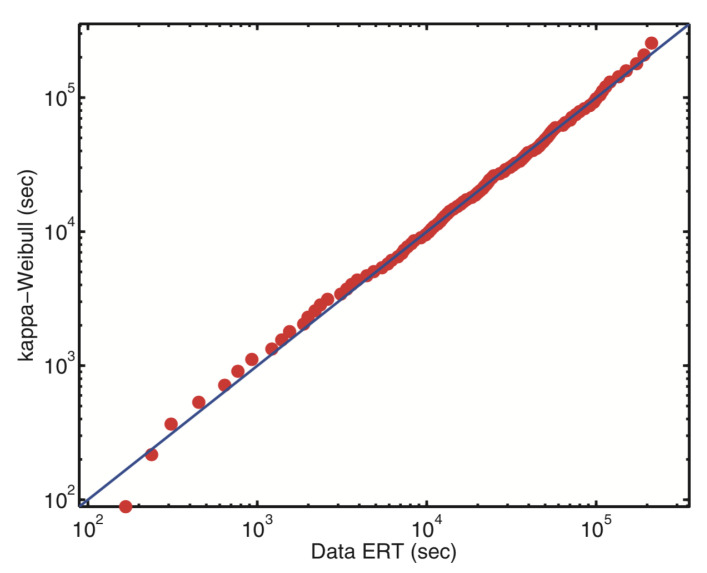
Quantiles associated with the distribution for the Cretan earthquake sequence return intervals (earthquake return times). The solid curve corresponds to the κ-Weibull distribution based on the κ-exponential function (Equation (12)), which, under appropriate constraints, extremizes the Kaniadakis κ-entropy. From [81].

This function satisfies $e_{\kappa }^{z}=e_{-\kappa }^{z}$, $e_{\kappa }^{z}e_{\kappa }^{-z}=1$ for all values of κ and has been used in the discussion of special relativity problems [25,26,27], among others. A successful application to earthquakes is illustrated in Figure 5, comparing the observed quantiles in Cretan seismic activity and those corresponding to an optimally fitted κ-Weibull distribution obtained by replacing, in the Weibull distribution, the exponential function by the κ-exponential function. We do not know how well other non-Boltzmannian entropies (Rényi, $S_{q}$, entropies yielding superstatistics) perform in this application. Such a focused comparison would naturally be enlightening and helpful.

Finally, to avoid some frequent confusion, let us mention that $z\propto x^{2}$ into $e_{\kappa }^{-z}$ is sometimes referred to as κ-Gaussian. This form should not be confused with the kappa distribution sometimes referred to as such in plasma physics, which was phenomenologically introduced half a century ago by Vasyliunas [82] and is in fact a *q*-Gaussian, *q* being simply related to the Vasyliunas κ index.

### 2.4. Beck–Cohen Superstatistics

**Figure 6:**
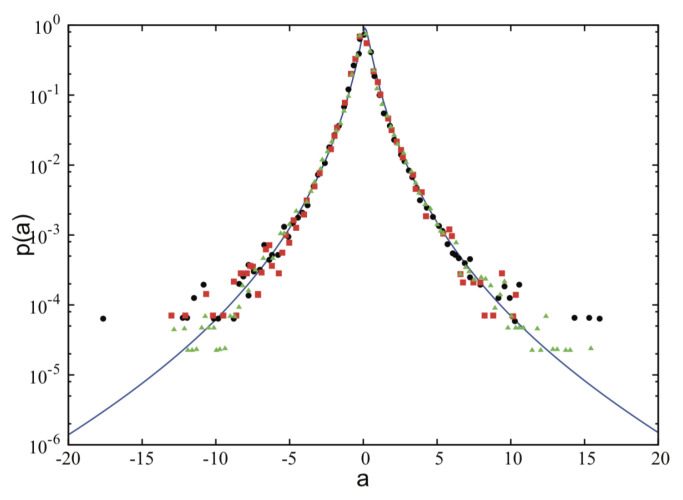
Experimental measurements of histograms of accelerations obtained by La Mantia et al. [86] and the Beck–Cohen lognormal superstatistics distribution (solid curve). From [87].

The BG basic weight is, of course, $e^{-\beta E}$, β being the inverse temperature of the system at thermal equilibrium. It happens, however, that for various reasons, the relevant stationary or quasi-stationary state of the system is one for which β fluctuates in space and/or time. The basic weight is then, in many cases, given by the deformed exponential

(13) $$e_{f}^{-\beta_{f}E}=\int_{0}^{\infty }d\beta f\left(\beta \right)e^{-\beta E}\;\;\;\;\;\left(\int_{0}^{\infty }d\beta f\left(\beta \right)=1\right)\;,$$

where f(β) represents the distribution of βs, to be determined analytically or experimentally from microscopic or mesoscopic dynamics. This approach was introduced by Beck and Cohen in 2003 [35], and since it represents a statistics within statistics, it has been named “superstatistics”. This simple and powerful formalism was inspired by the fact that when f(β) is the standard $\chi^{2}$ distribution, we verify straightforwardly [83,84] that $e_{f}^{-\beta_{f}E}=e_{q}^{-\beta_{q}E}$, thus recovering the stationary distribution of *q*-statistics. In the q→1 limit, f(β) corresponds to the standard Dirac delta distribution. The discrepancy with regard to the BG equilibrium state is measured by the Beck–Cohen index $q_{BC}=\langle {\left[f\left(\beta \right)\right]}^{2}\rangle /{\langle f\left(\beta \right)\rangle }^{2}$, which reproduces *q* for the $\chi^{2}$ superstatistics. Two other important cases correspond to 1/β following a $\chi^{2}$ distribution or β following a lognormal distribution; they are respectively referred to as inverse $\chi^{2}$ superstatistics and lognormal superstatistics. It has been shown [35] that for arbitrarily peaked f(β), the first-order correction with regard to the BG weight is always that of *q*-statistics, hence $0\;\leq q_{BC}-1\;∼q-1<<1$. The entropic functional whose appropriate extremization yields superstatistics is now available as well [34,85]. Superstatistics has been useful for a wide variety of systems. A successful application of lognormal superstatistics for quantum turbulence is depicted in Figure 6.

### 2.5. More Entropies and Applications

The BG entropy and its associated statistical mechanics have uncountable successful applications, which include very many in condensed matter classical and quantum systems, nuclear physics, and astrophysics, high-energy physics, theory of critical phenomena, among a plethora of other areas. However, when it comes to wide classes of complex systems—which may be nonergodic, weakly chaotic, long-range-interacting, long-memory-based—their theoretical approach usually becomes severely tricky, and more powerful concepts, such as nonadditive entropies, become extremely useful. In addition to those described above, various others have been advanced for a variety of reasons. To illustrate their mathematical diversity, let us display the precise analytical forms of some of them.

The nonadditive Borges–Roditi entropy is defined as [23]

(14) $$S_{q,q^{\prime }}^{BR}\equiv k\frac{\sum_{i=1}^{W}p_{i}^{q}-\sum_{i=1}^{W}p_{i}^{q^{\prime }}}{q^{\prime }-q}\;\;\;\;\left(S_{q,1}^{BR}=S_{1,q}^{BR}=S_{q}\right)\;.$$

The Curado entropy is defined as follows [30]:

(15) $$S_{b}^{C}\equiv k\left[\sum_{i=1}^{W}\left(1-e^{-bp_{i}}\right)+e^{-b}-1\right]\;\;\left(b\in R;b>0\right)\;.$$

Another exponential-form entropy was introduced in 2005 by Tsekouras and Tsallis [33]. It is defined as follows:

(16) $$S^{E}=k\sum_{i=1}^{W}p_{i}\left(1-e^{\frac{p_{i}-1}{p_{i}}}\right)\;,$$

where *E* stands for exponential. This entropic functional can be extended as follows:

(17) $$S_{c}^{E}=k\sum_{i=1}^{W}p_{i}\left(1-e^{c\frac{p_{i}-1}{p_{i}}}\right)\;\;\;\left(c\in R;c>0\right)\;.$$

The nonadditive Hanel–Thurner entropy, which has been determined by imposing specific asymptotic behaviors, is defined as [41] (see also [88])

(18) $$S_{c,d}^{HT}\equiv k\left[\frac{e\sum_{i=1}^{W}\Gamma \left(1+d,1-c\ln p_{i}\right)}{1-c+cd}-\frac{c}{1-c+cd}\right]\;\;\;\left(S_{q,0}^{HT}=S_{q}\right)\;,$$

where Γ denotes the incomplete gamma function. This two-parameter entropy has been been obtained by discussing the various asymptotic behaviors of W(N).

Let us also mention the nonadditive δ-entropy defined as follows [37], to be able to yield entropic extensivity for the stretched exponential dependence of *W* with *N* (see also [38,39]):

(19) $$S_{\delta }\equiv k\sum_{i=1}^{W}p{\left[\ln\frac{1}{p_{i}}\right]}^{\delta }\;\;\;\left(S_{1}=S_{BG}\right),$$

and its generalization, the entropy $S_{q,\delta }$ [40]:

(20) $$S_{q,\delta }\equiv k\sum_{i=1}^{W}p{\left[\ln_{q}\frac{1}{p_{i}}\right]}^{\delta }\;\;\;\left(S_{1,1}=S_{BG};\;S_{q,1}=S_{q};\;S_{1,\delta }=S_{\delta }\right).$$

The simple δ-entropy yields, for δ≠1, stretched exponential distributions, which are by no means rare in nature. Such distributions can also be obtained through the Anteneodo–Plastino entropy [32] and the Hanel–Thurner entropy [41]. The first physical applications of the δ entropy concern black holes [40] and cosmological evolution [89].

Let us end this section by mentioning other selected entropic applications beyond BG in physics: long-range interacting many-body classical Hamiltonian systems (XY model [90,91,92,93,94,95,96,97], Heisenberg model [98,99,100], Fermi–Pasta–Ulam (FPU) model [101,102,103,104]) (see [105,106] for earlier related approaches of the original FPU model and also [107], where the existence of non-Maxwellian compact-support momenta distributions are detected for special initial conditions); quantum-entangled low-dimensional Hamiltonian systems [108,109,110]; plasma physics [111,112,113,114,115]; turbulence [87,116]; astrophysics, cosmology, and black holes [89,117,118,119,120,121,122]; nonlinear dynamical systems [123,124,125,126,127,128]; nonlinear quantum mechanics [129,130,131,132]; anomalous diffusion, type II superconductors, and repulsive short-range interacting systems with overdamping [133,134,135,136,137]; hydrogen-like atoms [138,139]; viscous fingering [140]; glasses and spin-glasses [141]; astronomy and planetary physics (asteroids [142], meteor showers, and lunar flashes [143]); solar physics (solar wind [112,144]); generalized thermostatistics [145,146,147], among others [148].

Also worth mentioning are selected entropic applications beyond BG in other areas of knowledge: complex networks [16,17,18]; economics [149,150,151,152,153,154,155,156]; geophysics (earthquakes, atmosphere) [157,158,159,160,161,162,163,164,165,166]; general and quantum chemistry [139,167,168,169,170,171]; hydrology and engineering (water engineering [172] and materials engineering [173,174]); power grids [175]; the environment [176]; medicine [177,178,179]; biology [180,181]; computational processing of medical images (microcalcifications in mammograms [182] and magnetic resonance for multiple sclerosis [183]) and time series (e.g., ECG in coronary disease [184] and EEG in epilepsy [185,186]); train delays [187]; citations of scientific publications and scientometrics [188,189]; global optimization techniques [190,191,192]; ecology [193,194,195]; cognitive science [196,197,198]; mathematics (functions [199], uniqueness theorems and related axiomatic approaches [200,201,202,203,204,205], central limit theorems, and generalized Fourier transform [206,207,208,209,210,211,212,213,214]); probabilistic models [215,216,217]; information geometry [218,219].

Some of these applications beyond BG rely on the various available entropic forms; others directly rely on their optimizing distributions such as *q*-exponentials, κ-exponentials, and superstatistics. Some of these distributions have compact support (like *q*-exponentials with q<1); others have infinite support (like *q*-exponentials with q≥1).

Most of these applications concern $S_{q}$; some of them concern Rényi, Kaniadakis, and superstatistical approaches differing from *q*-statistics. To gain some deeper insight about this fact, it is convenient to focus on a few generic properties that play a relevant role outside the non-Boltzmannian world, namely additivity, trace-form, composability, and discriminative power.

An entropic functional is said of the trace-form if it can be written as $\sum_{i=1}^{W}f\left(p_{i}\right)$, where f(x) is some specific function. Therefore, the additive BG entropy is of the trace-form with f(x)=−kxlnx. The nonadditive *q*-entropy also satisfies this property with $f\left(x\right)=k\frac{x-x^{q}}{q-1}$, as does the nonadditive Kaniadakis entropy, as can be seen from its definition in Equation (11). In contrast, the additive Rényi entropy is, for q≠1, not of the trace form. Trace form certainly has value as a hypothesis of analytical simplicity, but it could, in principle, be violated without particularly heavy consequences, as far as we can tell.

An entropic functional is said composable if it verifies, for independent subsystems *A* and *B*, that S(A+B)/k=Φ[S(A)/k,S(B)/k], where Φ(x,y) is some function satisfying Φ(x,y)=Φ(y,x). This property has great thermodynamical relevance since it means that the macroscopic entropic value of a system composed of independent subsystems only depends on the macroscopic entropic values of the parts, and not on their microscopic details. Consequently, the BG and Rényi entropies are composable with Φ(x,y)=x+y, and the *q*-entropy is composable with Φ(x,y)=x+y+(1−q)xy. In contrast, the Kaniadakis entropy and many others are not composable; indeed, a linear combination of composable entropies is not necessarily composable.

By discriminative power, we mean the possibility of adaptation of the entropic functional to universal features of the system. Therefore, the BG entropy has no discriminative power at all since its definition involves no computable or fitting parameter (this might be considered as the “secret” reason for its wonderful success in very many systems over the last 140 years, but also for its weakness for attacking ubiquitous complex systems that have been intensively focused on in recent decades). The *q*-entropy and the Kaniadakis entropy both have discriminative power, respectively realized through their indices *q* and κ. The (q,δ)-entropy and the Hanel–Thurner one have even stronger discriminating power since they involve two indices, (q,δ) and (c,d), respectively. The entropic functionals yielding superstatistics have very strong discriminating power, represented by a full distribution [f(β)], as can be seen in Equation (13). The Rényi entropy has discriminating power (since it involves its index *q*), however weaker than those of the *q*-, Kaniadakis and superstatistical entropies. This weakness ultimately comes from the fact that in order to preserve the BG additivity for all values of *q*, its maximal value is necessarily universal, namely klnW, independently of the value of its index *q*. This restriction ends up decreasing its domain of useful applications. Its generic additivity also ends up decreasing its capability of producing thermodynamically extensive entropies for many complex systems, whose total number of admissible microscopic configurations increases non-exponentially with the number of particles or of degrees of freedom. As a final remark, let us mention that very many complex systems exhibit generic power laws in their properties, and the index *q* satisfactorily reflects the exponents of those power laws; by “generic”, we mean that the power laws emerge at large domains of variation of the external parameters acting on the system, in contrast with, say, traditional critical phenomena, where the power laws only appear at very specific values of the external parameters, whether they are tuned from the outside (like most second-order phase transitions) or self-tuned (like in self-organized criticality).

After deeper thinking involving all the elements described above, it is not particularly surprising that most of the applications beyond BG concern the *q*-entropy and *q*-statistics. Indeed, most of those applications, in physics and elsewhere, appear to accommodate satisfactorily with nonadditivity, trace-form, composability, and discriminating power, and $S_{q}$ happens to be the unique (see [204]) entropic functional simultaneously having all of those properties. As a mathematical curiosity, let us mention that all but two of the entropies mentioned in this review generalize that of BG. Both exceptions are of the exponential form, namely the Curado entropy [30,31] and the entropy (17), which do not recover the BG form for any of their particular instances. They therefore constitute not proper generalizations but rather alternative forms. As such, finding systems whose entropic description would require these specific entropic functionals remains an interesting challenge, especially due to their property that, for equal probabilities, they do not diverge for W→∞ but saturate instead, similarly to $S_{q}$ for q>1.

## 3. Further Connections

### 3.1. Thermodynamical Background

Some generic considerations are relevant at this point. Even when we face situations where many of the usual thermodynamical quantities scale with size in a non-traditional manner (for example, it is well known that microscopic long-range interacting two-body couplings in a conservative macroscopic system yield a superextensive internal energy), the extensivity of entropy must prevail. This is a nontrivial consequence of at least two interrelated reasons, namely to preserve in all cases the Legendre-transformation structure of classical thermodynamics [37,40] and to conform to some available illustrations for an extended form of the large deviation theory in the presence of long-range correlated random variables within a certain class [220,221,222].

**Figure 7:**
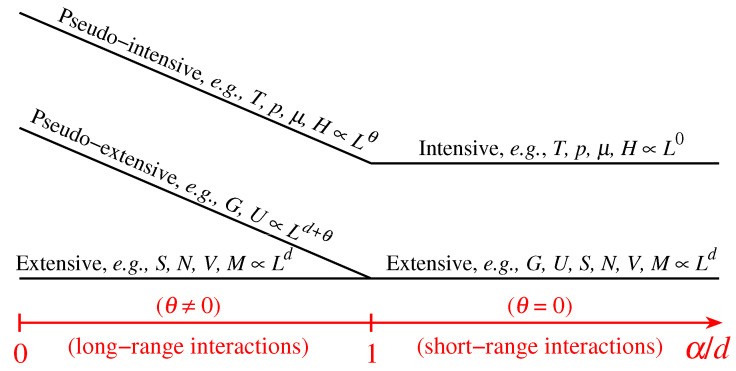
Representation of the different size-scaling regimes for classical *d*-dimensional systems. For attractive long-range interactions (i.e., 0≤α/d≤1, α characterizing the interaction range in a potential with the form $1/r^{\alpha }$; for example, Newtonian gravitation corresponds to (d,α)=(3,1)), we may distinguish three classes of thermodynamic variables, namely, those scaling with $L^{\theta }$, named pseudo-intensive (*L* is a characteristic linear length, θ is a system-dependent parameter), those scaling with $L^{d+\theta }$ with θ=d−α, the pseudo-extensive ones (the energies), and those scaling with $L^{d}$ (which are always extensive). For short-range interactions (i.e., α>d) we have θ=0, and the energies recover their standard $L^{d}$ extensive scaling, falling in the same class of *S*, *N*, *V*, etc., whereas the previous pseudo-intensive variables become truly intensive ones (independent of *L*); this is the region, with only two classes of variables, that is covered by the traditional textbooks of thermodynamics. From [37,40,50,223].

**Figure 8:**
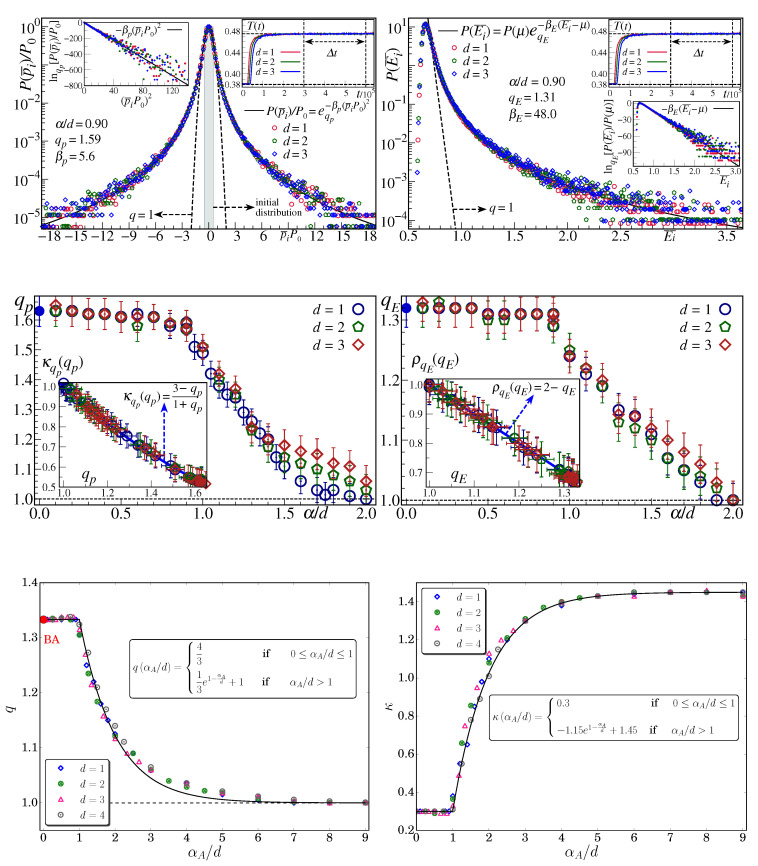
α-XY *d*-dimensional ferromagnet (for d=1,2,3). The time averages are done within the intervals Δt indicated in the insets. **Top left:** illustration of *q*-Gaussian fitting for the distribution of one-particle momentum (for comparison, the BG Gaussian is shown by a dashed line). **Top right:** illustration of *q*-exponential fitting for one-particle energy (for comparison, the BG weight is shown by a dashed line). **Middle left:** the α/d-dependence of the index $q_{p}$. **Middle right:** the α/d-dependence of the index $q_{E}$. We verify that above the critical value α/d=1, a region exists for which we numerically observe q>1. It cannot be excluded, at this stage, that this is not a consequence of the finiteness of the system size *N* and/or of the interval within which the time average is performed, and/or of the time *t* elapsed before starting the time average. Only (up to now intractable) analytical results or extremely heavy numerical calculations could definitively enlighten this complex region. It could, for example, happen that the relevant nontrivial results require simultaneously N→∞ and t→∞ along appropriately scaled paths. From [97]. Asymptotically scale-free *d*-dimensional network (for d=1,2,3,4). The distribution of degree *k* is well fitted by $P\left(k\right)=P\left(0\right)e_{q}^{-k/\kappa }$. **Bottom left:** the $\alpha_{A}/d$-dependence of the index *q*. The red dot indicates the Barabási–Albert (BA) universality class q=4/3 [19,22], which is here recovered as the $\alpha_{A}=0$ particular instance. **Bottom right:** the $\alpha_{A}/d$-dependence of the characteristic degree “temperature” κ. From [16]. In all cases, the BG (q=1) description naturally emerges numerically at α/d→∞ and even before.

To be concrete, let us assume that we have a classical many-body *d*-dimensional Hamiltonian with two-body interactions whose potential diverges at short distances and is attractive like $-1/r^{\alpha }$ at long distances. Its general thermodynamical energy *G* in its finite densitary form is given by [37,40,223]

(21) $$\begin{array}{c} \begin{array}{cc} \frac{G(V,T,p,\mu ,H,\ldots )}{L^{d+\theta }} & =\frac{U(V,T,p,\mu ,H,\ldots )}{L^{d+\theta }}-\frac{T}{L^{\theta }}\;\frac{S(V,T,p,\mu ,H,\ldots )}{L^{d}}+\\ + & \;\;\frac{p}{L^{\theta }}\;\frac{V}{L^{d}}-\frac{\mu }{L^{\theta }}\;\frac{N(V,T,p,\mu ,H,\ldots )}{L^{d}}-\frac{H}{L^{\theta }}\;\frac{M(V,T,p,\mu ,H,\ldots )}{L^{d}}-\ldots , \end{array} \end{array}$$

where *V* ($\equiv L^{d}$), T,p,μ,H are the volume, temperature, pressure, chemical potential and external magnetic field, and U,S,V,N,M are the internal energy, entropy, volume, number of particles and magnetization, respectively; L→∞ is the linear size of the system, and θ=d(1−α/d) (see Figure 7). As we can verify, the total entropy *S* belongs to the same thermodynamical class as (V,N,M) and is therefore extensive for arbitrary values of θ. A Hamiltonian system that belongs to the above scenario is the α-XY inertial ferromagnetic *d*-dimensional model [90]. Its non-Boltzmannian one-particle distributions of momenta and of energies are exhibited in Figure 8. In the same figure, we display the numerical results corresponding to an asymptotically scale-free *d*-dimensional network model [16]. We see here that both the thermal and the geometrical model exhibit the interesting α/d scaling. The same happens for the α-Heisenberg inertial ferromagnet [100], the α-Fermi–Pasta–Ulam model [101,102,103,104], and other asymptotically scale-free networks [17,18], thus exhibiting the ubiquity of this grounding scaling law.

Before proceeding, let us clarify why the statistical mechanical description of scale-free networks appears as a particular instance of *q*-statistics. If we associate each link with an effective “energy” ϵ, we may consider that ϵ/2 contributes to each of the two nodes connected by that link. Consequently, the total effective energy associated with each node is just half the sum of the effective energies corresponding to all the bonds arriving to that node. That node total energy is therefore proportional to the degree *k* of that node, and the energy of the entire system is the sum of all the node energies. We can then handle this system energy as a many-body Hamiltonian, and its generalized canonical distribution is therefore given by the *q*-exponential distribution of the form $e_{q}^{-k/\kappa }$ ($\propto 1/k^{\gamma }$ for k>>1, where γ≡1/(q−1)). Indeed, in the optimization of the *q*-entropy, the traditional internal energy constraint is replaced by the constraint on the mean value of the degree ϵ (i.e., *k*). Consequently, the quantity noted κ in Figure 8 plays precisely the role of an effective “temperature”. The size *N* of the network plays, in what concerns the stationary state, the same role as the number of particles of a many-body Hamiltonian (e.g., the number of rotators in the α-XY model, also illustrated in Figure 8). This shows that sentences such as “growth and preferential attachment are needed simultaneously to reproduce the stationary power-law distribution observed in real networks” [20] represent some sort of inadvertence. In contrast with preferential attachment, growth is not necessary. This is explicitly verified in the model discussed in [21], where neat numerical *q*-exponentials emerge for the degree distribution in spite of the fact that the model has a fixed (typically large) size.

Let us focus now on the intriguing facts illustrated at the middle (left and right) plots of Figure 8. The issue is why $q_{p}$ and $q_{E}$ seem to attain the BG regime (q=1) only for very large values of α/d and not from α/d=1 on. Indeed, the thermodynamical variables indicated in Equation (21), as well as the dynamical sensitivity to the initial conditions (more precisely, below what value of α/d the largest Lyapunov exponent vanishes [90,91]), strongly indicate α/d=1 as the frontier between the regions of validity of BG statistics and *q*-statistics for Hamiltonian systems. This important point still remains numerically elusive. The simulations are performed for finite size (*N*) systems and for finite time (*t*) evolution (in addition to other numerical-precision features related to the algorithm implemented to integrate the Newtonian differential equations). The mathematical validity of *q*-statistics is expected to require, due to subtle nonuniform convergences, the simultaneous limits (N,t)→(∞,∞), possibly not one after the other but simultaneous divergence along a scaling such as $t∼N^{\delta (\alpha /d)}$ with δ(α/d)>0 (even perhaps δ(α/d)>1). Such a scaling would imply that, for the increasingly large values of *N* (e.g., $N≃10^{6}$) that we computationally use, unaffordably large times *t* would be necessary before achieving the virtually stationary thermostatistical state of the system. In any case, for the entire range α/d≥0, *q*-Gaussians for the momenta distribution and *q*-exponentials for the energy distributions appear to be excellent approximations, as we can verify in the left and right insets of Figure 8 (middle), respectively, $\kappa_{q_{p}}\left(q_{p}\right)$ and $\rho_{q_{E}}\left(q_{E}\right)$.

In addition to the thermodynamical scalings discussed above in terms of the Legendre transform structure, another strong indication that the extensivity of the entropy must be preserved in all circumstances (even at the price of sacrificing, if necessary, the BG entropic additivity; analogously, the passage from Newtonian to Einstein mechanics in order to achieve a higher goal, namely the unification, through the Lorentz transform, of mechanics and Maxwell electromagnetism requires a small price, namely to sacrifice the simple Galilean additivity of velocities $v_{13}=v_{12}+v_{23}$ for one-dimensional motion, replacing it by the relativistic composition of velocities $v_{13}=\left(v_{12}+v_{23}\right)/(1+\frac{v_{12}}{c}\frac{v_{23}}{c}$)) is consistent with the numerical discussion of a nontrivial example within large deviation theory in the presence of long-range correlated random variables [220,221,222].

In the Table 1, we have summarised typical choices of entropic functionals corresponding to classes of non-vanishing-probability occupation of phase space for an increasing number of elements (or number of degrees of freedom) *N*.

### 3.2. q-Triplets

If we have a complex system characterized by some nonadditive entropy ($S_{q}$, $S_{c,d}^{HT}$, or any other), the entropic indices are expected to be different for essentially different properties (they are typically equal only for BG systems, i.e., for q=1). This fact has indeed been verified for *q*-systems, like solar wind [144], ozone layer [164], El Niño [165], and many others [156,166]. These various values for *q* appear to be isomorphic to the set of integer numbers; the central elements of this set constitute what is frequently referred to as the *q*-triplet. For a given system, only a small number of those values are apparently independent, all the others being related to those few through still only partially known analytic connections. Although some meaningful progress has been achieved [64,224], the full scenario constitutes an open problem, even if it is by now clear that it is quite similar to the critical phenomena classes of universality.

**Figure 9:**
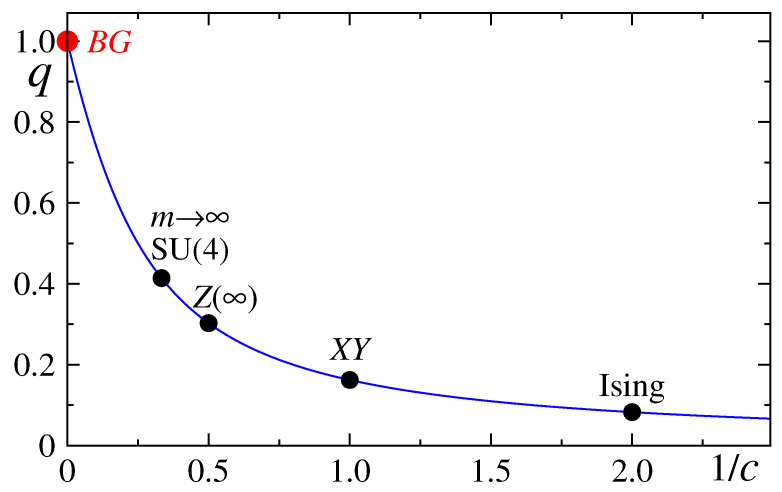
The index *q* has been determined [108] from first principles, namely from the universality class of the Hamiltonian. The values c=1/2 and c=1 respectively correspond to the Ising and XY ferromagnetic chains in the presence of a transverse field at T=0 criticality. For other models, see [225,226]. In the c→∞ limit, we recover the BG value, i.e., q=1. For arbitrary values of *c*, the subsystem nonadditive entropy $S_{q}$ is thermodynamically extensive for, and only for, $q\;=\;\frac{\sqrt{9+c^{2}}-3}{c}$ (hence $c=\frac{6q}{1-q^{2}}$; some special values: for c=4 we have q=1/2, and for c=6 we have $q\;=\;\frac{2}{\sqrt{5}+1}\;=\;\frac{1}{\Phi }$, where Φ is the golden mean). Let us emphasize that this anomalous value of *q* occurs only at precisely the zero-temperature second-order quantum critical point; anywhere else, the usual short-range-interaction BG behavior (i.e., q=1) is valid. From [227].

The set of values {q} is to be obtained from first-principle mechanics. However, such analytical calculations are frequently mathematically intractable. This is the only reason why in many examples of the literature, indices *q* are handled as fitting parameters. Still, some analytical calculations are nevertheless available, such as the case indicated in Figure 9.

## 4. Conclusions and Perspectives

We have argued that the additivity of the BG entropy accommodates extremely well, as is extensively known, to those many systems whose elements are generically not very strongly correlated and/or whose nonlinear dynamics are governed by strong chaos, meaning that their dynamics are associated to a sensitivity to initial conditions that exponentially diverge with time. But it fails for those complex systems that do not satisfy such requirements, violating, in particular, the thermodynamic extensivity expected (to satisfy the Legendre structure of classical thermodynamics and to yield an appropriately generalized theory of large deviations) for their entropy and whose dynamical sensitivity to initial conditions diverges sub-exponentially with time. In contrast, nonadditive entropies going beyond the BG entropy do solve these difficulties. This is consistent with Einstein’s 1910 remark [227,228] stating that the equal-probability BG entropy is the only reasonable one if we assume additivity, but he said nothing about additivity itself being necessary! (In fact, the Einstein requirement for likelihood factorization, an epistemologically sound principle, is valid for all values of *q*, a remarkable property related to the fact that the *q*-entropy is the unique trace-form composable entropy [204].) Notice also that if we do not require the trace form, the Rényi entropy also satisfies, for all values of *q*, the Einstein likelihood factorization principle. At this point, let us emphasize that the loss of entropic additivity is a rather small price to pay in order to not loose entropic extensivity, thus preserving thermodynamics and its Legendre-transform structure, in agreement with Einstein’s celebrated declaration [229].

Although many entropy-based theories are available in the literature that generalize or extend the BG form, those that have up to now provided neat and interesting applications in a plethora of natural, artificial, and social systems are restricted to a few of them. Those few include the *q*-entropy, Rényi entropy, Kaniadakis entropy, and Beck–Cohen superstatistics. Forms that repeatedly emerge in such applications, thus extending the BG exponential form, are *q*-exponentials, κ-exponentials, stretched exponentials, and Lambert functions. The utility of these forms in describing specific properties has been verified in wide classes of complex systems, including asymptotically scale-free networks.

Let us close by mentioning an ensemble of intriguing interrelated open problems that definitively enlarge, along different paths, the perspectives in both pure and applied sciences and technologies. We may cite the analytic dependencies on α/d of the *q* indices associated to the stationary (or quasi-stationary) time-averaged distributions of velocities and of energies, as well as their connection with the dynamical sensitivity to the initial conditions, in long-range interacting many-body classical Hamiltonian systems. Similarly, what characterizes the universality classes associated with low-dimensional conservative maps? Another central question that needs further progress is: What are the values of the various interrelated *q*-indices (to be obtained from first-principle mechanics, as exhibited in the few cases where the involved mathematics is tractable) corresponding to a given class of complex systems, and how many of those indices are expected to be independent [224]? In addition, it would be very valuable to extend the present *q*-central limit theorem [206] to q<1 values and to find the necessary and sufficient conditions for *q*-Gaussians and other deformed Gaussians (e.g., Kaniadakis κ-Gaussians, stretched exponentials, (q,α)-stable distributions [207]) to be attractors in the distribution space and analogously, in what concerns the large deviation theory directly related to *q*-exponential and possibly other deformed-exponential distributions. Another deep problem that needs further clarification is what controls the various known classes of superstatistics, including the necessary and sufficient a priori operational criteria that indicate the applicability, for a given system, of BG statistics, *q*-statistics, Kaniadakis κ-statistics, or something else. Such highly nontrivial questions have not yet been transparently elucidated, not even for BG statistical mechanics [230]!

## Figures and Tables

**Table 1:** Illustration of popular entropic functionals and the classes of systems to which they apply in order to exhibit thermodynamic extensivity. Additivity/nonadditivity only depends on the entropic functional, whereas extensivity/nonextensivity also depends on the system. An example of the power-law class can be seen in [64]. Notice that $S_{\delta }$ is not the only entropy to be consistent with extensivity for the stretched-exponential family; another possibility is $S_{c,d}^{HT}$ [41] with specific γ-dependent values for (c,d). It is important to also note that the behavior of *W* with *N* (N→∞) is not enough for uniquely determining the class of entropies yielding thermodynamic extensivity: the values here indicated for *q* and δ are valid under the strong hypothesis of equiprobability. To make this point transparent, we can check for the quantum critical point for a d=1 system characterized by the central charge $\bar{c}$ [108] that the entropy $S_{q}$ is extensive for $q=\left(\sqrt{9+{\bar{c}}^{2}}-3\right)/\bar{c}$. However, if we wrongly assume that the state corresponds to equiprobability, we obtain the wrong result $q=1-3/\bar{c}$. Both results recover the BG value q=1 for $1/\bar{c}\to 0$ but definitively differ for $1/\bar{c}>0$.

Systems	Entropy $S_{BG}$	Entropy $S_{q}$	Entropy $S_{\delta }$
W(N)		(q≠1)	(δ≠1)
(Equiprobable)	(Additive)	(Nonadditive)	(Nonadditive)
e.g., $\mu^{N}$	EXTENSIVE	NONEXTENSIVE	NONEXTENSIVE
(μ>1)			
e.g., $N^{\rho }$	NONEXTENSIVE	EXTENSIVE	NONEXTENSIVE
(ρ>0)		(q=1−1/ρ)	
e.g., $v^{N^{\gamma }}$	NONEXTENSIVE	NONEXTENSIVE	EXTENSIVE
(v>1;0<γ<1)			(δ=1/γ)

## References

[1] Boltzmann L. (1872). Weitere Studien uber das Wȧrmegleichgewicht unter Gas Molekulen [Further Studies on Thermal Equilibrium Between Gas Molecules].

[2] Boltzmann L., Sitzungsberichte K. (1877). Uber die Beziehung eines Allgemeine Mechanischen Satzes zum Zweiten Haupsatze der Warmetheorie.

[3] Gibbs J.W. (1902). Elementary Principles in Statistical Mechanics—Developed with Especial Reference to the Rational Foundation of Thermodynamics.

[4] Von Neumann J. (1927). Thermodynamik Quantenmechanischer Gesamtheiten.

[5] Shannon C.E. (1948). A Mathematical Theory of Communication. Bell Syst. Tech. J..

[6] Penrose O. (1970). Foundations of Statistical Mechanics: A Deductive Treatment.

[7] Renyi A. (1961). On measures of information and entropy. Proceedings of the Fourth Berkeley Symposium.

[8] Havrda J., Charvat F. (1967). Concept of Structural *α*-Entropy. Kybernetika.

[9] Sharma B.D., Mittal D.P. (1975). New non-additive measures of entropy for discrete probability distributions. J. Math. Sci..

[10] Tsallis C. (1988). Possible generalization of Boltzmann-Gibbs statistics. J. Stat. Phys..

[B11-entropy-21-00696] 11.In [3]: In treating of the canonical distribution, we shall always suppose the multiple integral in Equation (92) [the partition function, as we call it nowadays] to have a finite value, as otherwise the coefficient of probability vanishes, and the law of distribution becomes illusory. This will exclude certain cases, but not such apparently, as will affect the value of our results with respect to their bearing on thermodynamics. It will exclude, for instance, cases in which the system or parts of it can be distributed in unlimited space […]. It also excludes many cases in which the energy can decrease without limit, as when the system contains material points which attract one another inversely as the squares of their distances. […]. For the purposes of a general discussion, it is sufficient to call attention to the assumption implicitly involved in the Formula (92).

[B12-entropy-21-00696] 12.Fermi, E., *Thermodynamics*, (Dover, New York, 1936), page 53: The entropy of a system composed of several parts is very often equal to the sum of the entropies of all the parts. This is true if the energy of the system is the sum of the energies of all the parts and if the work performed by the system during a transformation is equal to the sum of the amounts of work performed by all the parts. Notice that these conditions are not quite obvious and that in some cases they may not be fulfilled. Thus, for example, in the case of a system composed of two homogeneous substances, it will be possible to express the energy as the sum of the energies of the two substances only if we can neglect the surface energy of the two substances where they are in contact. The surface energy can generally be neglected only if the two substances are not very finely subdivided; otherwise, it can play a considerable role.

[B13-entropy-21-00696] 13.Majorana, E., *The value of statistical laws in physics and social sciences*. The original manuscript in Italian was published by G. Gentile Jr. in *Scientia* **36**, 58 (1942), and was translated into English by R. Mantegna in 2005: This is mainly because entropy is an addditive quantity as the other ones. In other words, the entropy of a system composed of several independent parts is equal to the sum of entropy of each single part. […] Therefore one considers all possible internal determinations as equally probable. This is indeed a new hypothesis because the universe, which is far from being in the same state indefinitively, is subjected to continuous transformations. We will therefore admit as an extremely plausible working hypothesis, whose far consequences could sometime not be verified, that all the internal states of a system are a priori equally probable in specific physical conditions. Under this hypothesis, the statistical ensemble associated to each macroscopic state *A* turns out to be completely defined, 1938.

[14] Cartwright J. (2014). Roll over, Boltzmann. Phys. World.

[15] A Regularly Updated Bibliography. http://tsallis.cat.cbpf.br/biblio.htm.

[16] Brito S.G.A., da Silva L.R., Tsallis C. (2016). Role of dimensionality in complex networks. Sci. Rep..

[17] Nunes T.C., Brito S., da Silva L.R., Tsallis C. (2017). Role of dimensionality in preferential attachment growth in the Bianconi-Barabasi model. J. Stat. Mech..

[18] Brito S., Nunes T.C., da Silva L.R., Tsallis C. (2019). Scaling properties of *d*-dimensional complex networks. Phys. Rev. E.

[19] Barabási A.L., Albert R. (1999). Emergence of scaling in random networks. Science.

[20] Albert R., Barabási A.L. (2002). Statistical mechanics of complex networks. Rev. Mod. Phys..

[21] Thurner S., Tsallis C. (2005). Nonextensive aspects of self-organized scale-free gas-like networks. Europhys. Lett..

[22] Emmerich T., Bunde A., Havlin S. (2014). Structural and functional properties of spatially embedded scale-free networks. Phys. Rev. E.

[23] Borges E.P., Roditi I. (1998). A family of non-extensive entropies. Phys. Lett. A.

[24] Kaniadakis G. (2001). Non linear kinetics underlying generalized statistics. Phys. A.

[25] Kaniadakis G. (2002). Statistical mechanics in the context of special relativity. Phys. Rev. E.

[26] Kaniadakis G., Lissia M., Scarfone A.M. (2004). Deformed logarithms and entropies. Phys. A.

[27] Kaniadakis G. (2005). Statistical mechanics in the context of special relativity. II. Phys. Rev. E.

[28] Abe S. (1997). A note on the *q*-deformation theoretic aspect of the generalized entropies in nonextensive physics. Phys. Lett. A.

[29] Landsberg P.T., Vedral V. (1998). Distributions and channel capacities in generalized statistical mechanics. Phys. Lett. A.

[30] Curado E.M.F. (1999). General aspects of the thermodynamical formalism. Braz. J. Phys..

[31] Curado E.M.F., Nobre F.D. (2004). On the stability of analytic entropic forms. Phys. A.

[32] Anteneodo C., Plastino A.R. (1999). Maximum entropy approach to stretched exponential probability distributions. J. Phys. A.

[33] Tsekouras G.A., Tsallis C. (2005). Generalized entropy arising from a distribution of *q*-indices. Phys. Rev. E.

[34] Tsallis C., Souza A.M.C. (2003). Constructing a statistical mechanics for Beck-Cohen superstatistics. Phys. Rev. E.

[35] Beck C., Cohen E.G.D. (2003). Superstatistics. Phys. A.

[36] Schwammle V., Tsallis C. (2007). Two-parameter generalization of the logarithm and exponential functions and Boltzmann-Gibbs-Shannon entropy. J. Math. Phys..

[37] Tsallis C. (2009). Introduction to Nonextensive Statistical Mechanics-Approaching a Complex World.

[38] Shafee F. (2007). Lambert function and a new non-extensive form of entropy. IMA J. Appl. Math..

[39] Ubriaco M.R. (2009). Entropies based on fractional calculus. Phys. Lett. A.

[40] Tsallis C., Cirto L.J.L. (2013). Black hole thermodynamical entropy. Eur. Phys. J. C.

[41] Hanel R., Thurner S. (2011). A comprehensive classification of complex statistical systems and an axiomatic derivation of their entropy and distribution functions. Europhys. Lett..

[42] Tempesta P. (2011). Group entropies, correlation laws, and zeta functions. Phys. Rev. E.

[43] Jensen H.J., Tempesta P. (2018). Group entropies: From phase space geometry to entropy functionals via group theory. Entropy.

[44] Curado E.M.F., Tempesta P., Tsallis C. (2016). A new entropy based on a group-theoretical structure. Ann. Phys..

[45] Tempesta P. (2016). Beyond the Shannon-Khinchin formulation: The composability axiom and the universal-group entropy. Ann. Phys..

[46] Jensen H.J., Pazuki R.H., Pruessner G., Tempesta P. (2018). Statistical mechanics of exploding phase spaces: Ontic open systems. J. Phys. A: Math..

[47] Beck C., Schlogl F. (1993). Thermodynamics of Chaotic Systems.

[48] Zander C., Plastino A.R., Casas M., Plastino A. (2012). Entropic entanglement criteria for Fermion systems. Eur. Phys. J. D.

[49] Tsallis C., Lloyd S., Baranger M. (2001). Peres criterion for separability through nonextensive entropy. Phys. Rev. A.

[50] Tsallis C. (2016). Approach of complexity in nature: Entropic nonuniqueness. Axioms.

[51] Curado E.M.F., Tsallis C. (1992). Generalized statistical mechanics: Connection with thermodynamics. J. Phys. A.

[52] Tsallis C., Mendes R.S., Plastino A.R. (1998). The role of constraints within generalized nonextensive statistics. Phys. A.

[53] Bediaga I., Curado E.M.F., Miranda J. (2000). A nonextensive thermodynamical equilibrium approach in *e*^+^*e*^−^ → *hadrons*. Phys. A.

[54] Adare A., Afanasiev S., Aidala C., Ajitanand N.N., Akiba Y., Al-Bataineh H., Alexander J., Aoki K., Aphecetche L., Armendariz R. (2011). (PHENIX Collaboration). Measurement of neutral mesons in *p* + *p* collisions at $\sqrt{s}=200\;\mathrm{GeV}$ and scaling properties of hadron production. Phys. Rev. D.

[55] ALICE Collaboration (2017). Enhanced production of multi-strange hadrons in high-multiplicity proton-proton collisions. Nat. Phys..

[56] Wong C.Y., Wilk G., Cirto L.J.L., Tsallis C. (2015). From QCD-based hard-scattering to nonextensive statistical mechanical descriptions of transverse momentum spectra in high-energy *pp* and $p\bar{p}$ collisions. Phys. Rev. D.

[57] Marques L., Cleymans J., Deppman A. (2015). Description of high-energy *pp* collisions using Tsallis thermodynamics: Transverse momentum and rapidity distributions. Phys. Rev. D.

[58] ALICE Collaboration (2017). Production of *π*^0^ and *η* mesons up to high transverse momentum in pp collisions at 2.76 TeV. Eur. Phys. J. C.

[59] ALICE Collaboration (2017). Production of Σ(1385)^±^ and Ξ(1530)^0^ in p-Pb collisions at $\sqrt{s_{NN}}=5.02\;\mathrm{TeV}$. Eur. Phys. J. C.

[60] ALICE Collaboration (2017). *K**(892)^0^ and Φ(1020) meson production at high transverse momentum in pp and Pb-Pb collisions at $\sqrt{s_{NN}}=2.76\;\mathrm{TeV}$. Phys. Rev. C.

[61] Rybczynski M., Wilk G., Wlodarczyk Z. (2015). System size dependence of the log-periodic oscillations of transverse momentum spectra. Eur. Phys. J. Web Conf..

[62] Wilk G., Wlodarczyk Z. (2015). Tsallis distribution decorated with log-periodic oscillation. Entropy.

[63] Yalcin G.C., Beck C. (2018). Generalized statistical mechanics of cosmic rays: Application to positron-electron spectral indices. Sci. Rep..

[64] Tsallis C., Gell-Mann M., Sato Y. (2005). Asymptotically scale-invariant occupancy of phase space makes the entropy *S_q_* extensive. Proc. Natl. Acad. Sci. USA.

[65] Plastino A.R., Plastino A. (1995). Non-extensive statistical mechanics and generalized Fokker-Planck equation. Phys. A.

[66] Schwammle V., Nobre F.D., Curado E.M.F. (2007). Consequences of the H-theorem from nonlinear Fokker-Planck equations. Phys. Rev. E.

[67] Tsallis C., Bukman D.J. (1996). Anomalous diffusion in the presence of external forces: Exact time-dependent solutions and their thermostatistical basis. Phys. Rev. E.

[68] Upadhyaya A., Rieu J.-P., Glazier J.A., Sawada Y. (2001). Anomalous diffusion and non-Gaussian velocity distribution of Hydra cells in cellular aggregates. Phys. A.

[69] Rapisarda A., Pluchino A. (2005). Nonextensive thermodynamics and glassy behavior. Europhys. News.

[70] Combe G., Richefeu V., Stasiak M., Atman A.P.F. (2015). Experimental validation of nonextensive scaling law in confined granular media. Phys. Rev. Lett..

[71] Viallon-Galiner L., Combe G., Richefeu V., Atman A.P.F. (2018). Emergence of shear bands in confined granular systems: Singularity of the *q*-statistics. Entropy.

[72] Lutz E. (2003). Anomalous diffusion and Tsallis statistics in an optical lattice. Phys. Rev. A.

[73] Douglas P., Bergamini S., Renzoni F. (2006). Tunable Tsallis distributions in dissipative optical lattices. Phys. Rev. Lett..

[74] Lutz E., Renzoni F. (2013). Beyond Boltzmann-Gibbs statistical mechanics in optical lattices. Nat. Phys..

[75] Tirnakli U., Tsallis C., Beck C. (2009). A closer look at time averages of the logistic map at the edge of chaos. Phys. Rev. E.

[76] Tsallis C., Plastino A.R., Zheng W.-M. (1997). Power-law sensitivity to initial conditions—New entropic representation. Chaos Solitons Fractals.

[77] Lyra M.L., Tsallis C. (1998). Nonextensivity and multifractality in low-dimensional dissipative systems. Phys. Rev. Lett..

[78] Baldovin F., Robledo A. (2004). Nonextensive Pesin identity. Exact renormalization group analytical results for the dynamics at the edge of chaos of the logistic map. Phys. Rev. E.

[79] Mayoral E., Robledo A. (2005). Tsallis’ *q* index and Mori’s *q* phase transitions at edge of chaos. Phys. Rev. E.

[80] Tirnakli U., Borges E.P. (2016). The standard map: From Boltzmann-Gibbs statistics to Tsallis statistics. Sci. Rep..

[81] Hristopulos D.T., Petrakis M.P., Kaniadakis G. (2014). Finite-size effects on return interval distributions for weakest-link-scaling systems. Phys. Rev. E.

[82] Vasyliunas V.M. (1968). A survey of low-energy electrons in the evening sector of the magnetosphere with OGO 1 and OGO 3. J. Geophys. Res..

[83] Wilk G., Wlodarczyk Z. (2000). Interpretation of the nonextensivity parameter *q* in some applications of Tsallis statistics and Levy distributions. Phys. Rev. Lett..

[84] Beck C. (2001). Dynamical foundations of nonextensive statistical mechanics. Phys. Rev. Lett..

[85] Hanel R., Thurner S., Gell-Mann M. (2011). Generalized entropies and the transformation group of superstatistics. Proc. Natl. Acad. Sci. USA.

[86] la Mantia M., Duda D., Rotter M., Skrbek L. (2013). Lagrangian accelerations of particles in superfluid turbulence. J. Fluid Mech..

[87] Miah S., Beck C. (2014). Lagrangian quantum turbulence model based on alternating superfluid/normal fluid stochastic dynamics. Europhys. Lett..

[88] Korbel J., Hanel R., Thurner S. (2018). Classification of complex systems by their sample-space scaling exponents. New J. Phys..

[89] Komatsu N., Kimura S. (2013). Entropic cosmology for a generalized black-hole entropy. Phys. Rev. D.

[90] Anteneodo C., Tsallis C. (1998). Breakdown of the exponential sensitivity to the initial conditions: Role of the range of the interaction. Phys. Rev. Lett..

[91] Campa A., Giansanti A., Moroni D., Tsallis C. (2001). Classical spin systems with long-range interactions: Universal reduction of mixing. Phys. Lett. A.

[92] Antoni M., Ruffo S. (1995). Clustering and relaxation in Hamiltonian long-range dynamics. Phys. Rev. E.

[93] Pluchino A., Rapisarda A., Tsallis C. (2007). Nonergodicity and central limit behavior in long-range Hamiltonians. Europhys. Lett..

[94] Pluchino A., Rapisarda A. (2007). Nonergodicity and central limit behavior for systems with long-range interactions. SPIE.

[95] Chavanis P.H., Campa A. (2010). Inhomogeneous Tsallis distributions in the HMF model. Eur. Phys. J. B.

[96] Cirto L.J.L., Assis V.R.V., Tsallis C. (2014). Influence of the interaction range on the thermostatistics of a classical many-body system. Phys. A.

[97] Cirto L.J.L., Rodriguez A., Nobre F.D., Tsallis C. (2018). Validity and failure of the Boltzmann weight. Europhys. Lett..

[98] Nobre F.D., Tsallis C. (2003). Classical infinite-range-interaction Heisenberg ferromagnetic model: Metastability and sensitivity to initial conditions. Phys. Rev. E.

[99] Cirto L.J.L., Lima L.S., Nobre F.D. (2015). Controlling the range of interactions in the classical inertial ferromagnetic Heisenberg model: Analysis of metastable states. JSTAT.

[100] Rodriguez A., Nobre F.D., Tsallis C. (2019). *d*-Dimensional classical Heisenberg model with arbitrarily-ranged interactions: Lyapunov exponents and distributions of momenta and energies. Entropy.

[101] Christodoulidi H., Tsallis C., Bountis T. (2014). Fermi-Pasta-Ulam model with long-range interactions: Dynamics and thermostatistics. EPL.

[102] Christodoulidi H., Bountis T., Tsallis C., Drossos L. (2016). Dynamics and statistics of the Fermi–Pasta–Ulam *β*–model with different ranges of particle interactions. J. Stat. Mech..

[103] Bagchi D., Tsallis C. (2016). Sensitivity to initial conditions of *d*-dimensional long-range-interacting quartic Fermi-Pasta-Ulam model: Universal scaling. Phys. Rev. E.

[104] Bagchi D., Tsallis C. (2018). Fermi-Pasta-Ulam-Tsingou problems: Passage from Boltzmann to *q*-statistics. Phys. A.

[105] Leo M., Leo R.A., Tempesta P. (2010). Thermostatistics in the neighborhood of the *π*-mode solution for the Fermi-Pasta-Ulam *β* system: From weak to strong chaos. J. Stat. Mech..

[106] Antonopoulos C.G., Bountis T.C., Basios V. (2011). Quasi-stationary chaotic states in multi-dimensional Hamiltonian systems. Phys. A.

[107] Leo M., Leo R.A., Tempesta P., Tsallis C. (2012). Non Maxwellian behaviour and quasi-stationary regimes near the modal solutions of the Fermi-Pasta-Ulam *β*-system. Phys. Rev. E.

[108] Caruso F., Tsallis C. (2008). Nonadditive entropy reconciles the area law in quantum systems with classical thermodynamics. Phys. Rev. E.

[109] Saguia A., Sarandy M.S. (2010). Nonadditive entropy for random quantum spin-*S* chains. Phys. Lett. A.

[110] Carrasco J.A., Finkel F., Gonzalez-Lopez A., Rodriguez M.A., Tempesta P. (2016). Generalized isotropic Lipkin-Meshkov-Glick models: Ground state entanglement and quantum entropies. J. Stat. Mech..

[111] Lourek I., Tribeche M. (2016). On the role of the *κ*-deformed Kaniadakis distribution in nonlinear plasma waves. Phys. A.

[112] Bacha M., Gougam L.A., Tribeche M. (2017). Ion-acoustic rogue waves in magnetized solar wind plasma with nonextensive electrons. Phys. A.

[113] Merriche A., Tribeche M. (2017). Electron-acoustic rogue waves in a plasma with Tribeche-Tsallis-Cairns distributed electrons. Ann. Phys..

[114] Livadiotis G. (2018). Thermodynamic origin of kappa distributions. EPL.

[115] Oliveira D.S., Galvao R.M.O. (2018). Non-extensive transport equations in magnetized plasmas for non-Maxwellian distribution functions. Phys. Plasmas.

[116] Daniels K.E., Beck C., Bodenschatz E. (2004). Defect turbulence and generalized statistical mechanics. Phys. D.

[117] Komatsu N., Kimura S. (2014). Evolution of the universe in entropic cosmologies via different formulations. Phys. Rev. D.

[118] Komatsu N. (2017). Cosmological model from the holographic equipartition law with a modified Renyi entropy. Eur. Phys. J. C.

[119] Beck C. (2016). Cosmological flux noise and measured noise power spectra in SQUIDs. Sci. Rep..

[120] Hou S.Q., He J.J., Parikh A., Kahl D., Bertulani C.A., Kajino T., Mathews G.J., Zhao G. (2017). Non-extensive statistics solution to the cosmological Lithium problem. Astrophys. J..

[121] Kohler S. (2017). Fixing the Big Bang theory’s Lithium problem. NOVA—Res. Highlights J. Am. Astron. Soc..

[122] Bertulani C.A., Shubhchintak, Mukhamedzhanov A.M. (2018). Cosmological Lithium problems. EPJ Web Conf..

[123] Ruiz G., Tsallis C. (2007). Roundoff-induced attractors and reversibility in conservative two-dimensional maps. Phys. A.

[124] Ruiz G., Tsallis C. (2009). Nonextensivity at the edge of chaos of a new universality class of one-dimensional unimodal dissipative maps. Eur. Phys. J. B.

[125] Ruiz G., Bountis T., Tsallis C. (2012). Time-evolving statistics of chaotic orbits of conservative maps in the context of the Central Limit Theorem. Int. J. Bifurc. Chaos.

[126] Bountis T., Skokos H. (2012). Complex Hamiltonian Dynamics.

[127] Ruiz G., Tirnakli U., Borges E.P., Tsallis C. (2017). Statistical characterization of the standard map. J. Stat. Mech..

[128] Ruiz G., Tirnakli U., Borges E.P., Tsallis C. (2017). Statistical characterization of discrete conservative systems: The web map. Phys. Rev. E.

[129] Nobre F.D., Rego-Monteiro M.A., Tsallis C. (2011). Nonlinear generalizations of relativistic and quantum equations with a common type of solution. Phys. Rev. Lett..

[130] Filho R.N.C., Alencar G., Skagerstam B.-S., Andrade J.S. (2013). Morse potential derived from first principles. Europhys. Lett..

[131] Nobre F.D., Plastino A.R. (2017). A family of nonlinear Schrodinger equations admitting *q*-plane wave. Phys. Lett. A.

[132] Plastino A.R., Wedemann R.S. (2017). Nonlinear wave equations related to nonextensive thermostatistics. Entropy.

[133] Andrade J.S., da Silva G.F.T., Moreira A.A., Nobre F.D., Curado E.M.F. (2010). Thermostatistics of overdamped motion of interacting particles. Phys. Rev. Lett..

[134] Vieira C.M., Carmona H.A., Andrade J.S., Moreira A.A. (2016). General continuum approach for dissipative systems of repulsive particles. Phys. Rev. E.

[135] Zand J., Tirnakli U., Jensen H.J. (2015). On the relevance of *q*-distribution functions: The return time distribution of restricted random walker. J. Phys. A: Math..

[136] Curado E.M.F., Souza A.M.C., Nobre F.D., Andrade R.F.S. (2014). Carnot cycle for interacting particles in the absence of thermal noise. Phys. Rev. E.

[137] Nobre F.D., Curado E.M.F., Souza A.M.C., Andrade R.F.S. (2015). Consistent thermodynamic framework for interacting particles by neglecting thermal noise. Phys. Rev. E.

[138] Puertas-Centeno D., Temme N.M., Toranzo I.V., Dehesa J.S. (2017). Entropic uncertainty measures for large dimensional hydrogenic systems. J. Math. Phys..

[139] Vignat C., Plastino A., Plastino A.R., Dehesa J.S. (2012). Quantum potentials with *q*-Gaussian ground states. Phys. A.

[140] Grosfils P., Boon J.P. (2006). Nonextensive statistics in viscous fingering. Phys. A.

[141] Pickup R.M., Cywinski R., Pappas C., Farago B., Fouquet P. (2009). Generalized spin glass relaxation. Phys. Rev. Lett..

[142] Betzler A.S., Borges E.P. (2012). Nonextensive distributions of asteroid rotation periods and diameters. Astron. Astrophys..

[143] Betzler A.S., Borges E.P. (2015). Nonextensive statistical analysis of meteor showers and lunar flashes. Mon. Not. R. Astron. Soc..

[144] Burlaga L.F., -Vinas A.F. (2005). Triangle for the entropic index *q* of non-extensive statistical mechanics observed by Voyager 1 in the distant heliosphere. Phys. A.

[145] Naudts J. (2011). Generalised Thermostatistics.

[146] Biro T.S., Barnafoldi G.G., Van P. (2013). Quark-gluon plasma connected to finite heat bath. Eur. Phys. J. A.

[147] Bagci G.B., Oikonomou T. (2016). Validity of the third law of thermodynamics for the Tsallis entropy. Phys. Rev. E.

[148] Gell-Mann M., Tsallis C. (2004). Nonadditive Entropy—Interdisciplinary Applications.

[149] Borland L. (2002). Closed form option pricing formulas based on a non-Gaussian stock price model with statistical feedback. Phys. Rev. Lett..

[150] Borland L. (2002). A theory of non-gaussian option pricing. Quant. Financ..

[151] Kwapien J., Drozdz S. (2012). Physical approach to complex systems. Phys. Rep..

[152] Ruiz G., de Marcos A.F. (2018). Evidence for criticality in financial data. Eur. Phys. J. B.

[153] Borland L. (2017). Financial market models. Complexity and Synergetics.

[154] Xu D., Beck C. (2017). Symbolic dynamics techniques for complex systems: Application to share price dynamics. Europhys. Lett..

[155] Tsallis C. (2017). Economics and finance: *q*-statistical features galore. Entropy.

[156] Stosic D., Stosic D., Ludermir T.B., Stosic T. (2018). Nonextensive triplets in cryptocurrency exchanges. Phys. A.

[157] Yalcin G.C., Rabassa P., Beck C. (2016). Extreme event statistics of daily rainfall: Dynamical systems approach. J. Phys. A.

[158] Pavlos G.P., Karakatsanis L.P., Iliopoulos A.C., Pavlos E.G., Tsonis A.A. (2017). Non-extensive statistical mechanics: Overview of theory and applications in seismogenesis, climate, and space plasma. Advances in Nonlinear Geosciences.

[159] Bakar B., Tirnakli U. (2009). Analysis of self-organized criticality in Ehrenfest’s dog-flea model. Phys. Rev. E.

[160] Bakar B., Tirnakli U. (2010). Return distributions in dog-flea model revisited. Phys. A.

[161] Celikoglu A., Tirnakli U., Queiros S.M.D. (2010). Analysis of return distributions in the coherent noise model. Phys. Rev. E.

[162] Vallianatos F. (2018). A Non-extensive statistical mechanics view on Easter island seamounts volume distribution. Geosciences.

[163] Carbone F., Bruno A.G., Naccarato A., de Simone F., Gencarelli C.N., Sprovieri F., Hedgecock I.M., Landis M.S., Skov H., Pfaffhuber K.A. (2018). The superstatistical nature and interoccurrence time of atmospheric mercury concentration fluctuations. J. Geophys. Res..

[164] Ferri G.L., Savio M.F.R., Plastino A. (2010). Tsallis’ *q*-triplet and the ozone layer. Phys. A.

[165] Ferri G.L., Figliola A., Rosso O.A. (2012). Tsallis’ statistics in the variability of El Niño/Southern Oscillation during the Holocene epoch. Phys. A.

[166] Pavlos G.P., Karakatsanis L.P., Xenakis M.N., Sarafopoulos D., Pavlos E.G. (2012). Tsallis statistics and magnetospheric self-organization. Phys. A.

[167] Amador C.H.S., Zambrano L.S. (2010). Evidence for energy regularity in the Mendeleev periodic table. Phys. A.

[168] Morais S.F.D.A., Mundim K.C., Ferreira D.A.C. (2015). An alternative interpretation of the ultracold methylhydroxycarbene rearrangement mechanism: Cooperative effects. Phys. Chem. Chem. Phys..

[169] Sekania M., Appelt W.H., Benea D., Ebert H., Vollhardt D., Chioncel L. (2018). Scaling behavior of the Compton profile of alkali metals. Phys. A.

[170] Aquilanti V., Coutinho N.D., Carvalho-Silva V.H. (2017). Kinetics of low-temperature transitions and a reaction rate theory from non-equilibrium distributions. Philos. Trans. R. Soc. A.

[171] Aquilanti V., Borges E.P., Coutinho N.D., Mundim K.C., Carvalho-Silva V.H. (2018). From statistical thermodynamics to molecular kinetics: The change, the chance and the choice. The Quantum World of Molecules. Rendiconti Lincei.

[172] Singh V.P. (2016). Introduction to Tsallis Entropy Theory in Water Engineering.

[173] Stavrakas I., Triantis D., Kourkoulis S.K., Pasiou E.D., Dakanali I. (2016). Acoustic emission analysis of cement mortar specimens during three point bending tests. Lat. Am. Journ. Sol. Struct..

[174] Aifantis E.C. (2017). Towards internal length gradient chemomechanics. Rev. Adv. Mater. Sci..

[175] Schafer B., Beck C., Aihara K., Witthaut D., Timme M. (2018). Non-Gaussian power grid frequency fluctuations characterized by Levy-stable laws and superstatistics. Nat. Energy.

[176] Yalcin G.C., Beck C. (2013). Environmental superstatistics. Phys. A.

[177] Hagiwara Y., Sudarshan V.K., Leong S.S., Vijaynanthan A., Ng K.H. (2017). Application of entropies for automated diagnosis of abnormalities in ultrasound images: A review. J. Mech. Med. Biol..

[178] Tsigelny I.F., Kouznetsova V.L., Sweeney D.E., Wu W., Bush K.T., Nigam S.K. (2008). Analysis of metagene portraits reveal distinct transitions during kidney organogenesis. Sci. Signal..

[179] Sotolongo-Grau O., Rodriguez-Perez D., Antoranz J.C., Sotolongo-Costa O. (2010). Tissue radiation response with maximum Tsallis entropy. Phys. Rev. Lett..

[180] Bogachev M.I., Kayumov A.R., Bunde A. (2014). Universal internucleotide statistics in full genomes: A footprint of the DNA structure and packaging?. PLoS ONE.

[181] Bogachev M.I., Markelov O.A., Kayumov A.R., Bunde A. (2017). Superstatistical model of bacterial DNA architecture. Sci. Rep..

[182] Mohanalin J., Beenamol, Kalra P.K., Kumar N. (2010). A novel automatic microcalcification detection technique using Tsallis entropy and a type II fuzzy index. Comput. Math. Appl..

[183] Diniz P.R.B., Murta L.O., Brum D.G., de Araujo D.B., Santos A.C. (2010). Brain tissue segmentation using *q*-entropy in multiple sclerosis magnetic resonance images. Braz. J. Med Biol. Res..

[184] Acharya U.R., Hagiwara Y., Koh J.E.W., Oh S.L., Tan J.H., Adam M., Tan R.S. (2018). Entropies for automated detection of coronary artery disease using ECG signals: A review. Biocybern. Biomed. Eng..

[185] Capurro A., Diambra L., Lorenzo D., Macadar O., Martins M.T., Mostaccio C., Plastino A., Perez J., Rofman E., Torres M.E. (1999). Human dynamics: The analysis of EEG signals with Tsallis information measure. Phys. A.

[186] Acharya U.R., Hagiwara Y., Deshpande S.N., Suren S., Koh J.E.W., Oh S.L., Arunkumar N., Ciaccio E.J., Lim C.M. (2018). Characterization of focal EEG signals: A review. Future Gener. Comput. Syst..

[187] Briggs K., Beck C. (2007). Modelling train delays with *q*-exponential functions. Phys. A.

[188] Picoli S., Mendes R.S., Malacarne L.C., Lenzi E.K. (2006). Scaling behavior in the dynamics of citations to scientific journals. Europhys. Lett..

[189] Anastasiadis A.D., de Albuquerque M.P., de Albuquerque M.P., Mussi D.B. (2010). Tsallis *q*-exponential describes the distribution of scientific citations—A new characterization of the impact. Scientometrics.

[190] Tsallis C., Stariolo D.A. (1996). Generalized simulated annealing. Phys. A.

[191] Boulle A., Debelle A. (2010). Strain-profile determination in ion-implanted single crystals using generalized simulated annealing. J. Appl. Crystallogr..

[192] Shang C., Wales D.J. (2014). Communication: Optimal parameters for basin-hopping global optimization based on Tsallis statistics. J. Chem. Phys..

[193] Evangelista H.B.A., Thomaz S.M., Mendes R.S., Evangelista L.R. (2009). Generalized entropy indices to measure *α*- and *β*-diversities of macrophytes. Braz. J. Phys..

[194] Harte J., Newman E.A. (2014). Maximum information entropy: A foundation for ecological theory. Trends Ecol. Evol..

[195] Puzachenko Y.G. (2016). Rank Distribution in Ecology and Nonextensive Statistical Mechanics.

[196] Hadzibeganovic T., Cannas S.A. (2009). A Tsallis’ statistics based neural network model for novel word learning. Phys. A.

[197] Takahashi T., Hadzibeganovic T., Cannas S.A., Makino T., Fukui H., Kitayama S. (2009). Cultural neuroeconomics of intertemporal choice. Neuroendocrinol. Lett..

[198] Siddiqui M., Wedemann R.S., Jensen H. (2018). Avalanches and generalized memory associativity in a network model for conscious and unconscious mental functioning. Phys. A.

[199] Borges E.P. (1998). On a *q*-generalization of circular and hyperbolic functions. J. Phys. A.

[200] Santos R.J.V.d. (1997). Generalization of Shannon’s theorem for Tsallis entropy. J. Math. Phys..

[201] Abe S. (2000). Axioms and uniqueness theorem for Tsallis entropy. Phys. Lett. A.

[202] Boghosian B.M., Love P.J., Coveney P.V., Karlin I.V., Succi S., Yepez J. (2003). Galilean-invariant lattice-Boltzmann models with *H*-theorem. Phys. Rev. E.

[203] Tsallis C. (2015). Conceptual inadequacy of the Shore and Johnson axioms for wide classes of complex systems. Entropy.

[204] Enciso A., Tempesta P. (2017). Uniqueness and characterization theorems for generalized entropies. arXiv.

[205] Jizba P., Korbel J. (2019). Maximum entropy principle in statistical inference: Case for non-Shannonian entropies. Phys. Rev. Lett..

[206] Umarov S., Tsallis C., Steinberg S. (2008). On a *q*-central limit theorem consistent with nonextensive statistical mechanics. Milan J. Math..

[207] Umarov S., Tsallis C., Gell-Mann M., Steinberg S. (2010). Generalization of symmetric *α*-stable Lévy distributions for *q* > 1. J. Math. Phys..

[208] Hilhorst H.J. (2010). Note on a *q*-modified central limit theorem. J. Stat. Mech..

[209] Jauregui M., Tsallis C. (2011). *q*-generalization of the inverse Fourier transform. Phys. Lett. A.

[210] Jauregui M., Tsallis C., Curado E.M.F. (2011). *q*-moments remove the degeneracy associated with the inversion of the *q*-Fourier transform. J. Stat. Mech..

[211] Plastino A., Rocca M.C. (2012). Inversion of Umarov-Tsallis-Steinberg’s *q*-Fourier Transform and the complex-plane generalization. Phys. A.

[212] Plastino A., Rocca M.C. (2012). *q*-Fourier Transform and its inversion-problem. Milan J. Math..

[213] Umarov S., Tsallis C. (2016). The limit distribution in the *q*-CLT for *q* ≥ 1 is unique and can not have a compact support. J. Phys. A.

[214] Sicuro G., Tsallis C. (2017). *q*-Generalized representation of the d-dimensional Dirac delta and *q*-Fourier transform. Phys. Lett. A.

[215] Rodriguez A., Schwammle V., Tsallis C. (2008). Strictly and asymptotically scale-invariant probabilistic models of *N* correlated binary random variables having *q*-Gaussians as *N* → ∞ limiting distributions. J. Stat. Mech..

[216] Ruiz G., Tsallis C. (2015). Emergence of *q*-statistical functions in a generalized binomial distribution with strong correlations. J. Math. Phys..

[217] Bergeron H., Curado E.M.F., Gazeau J.P., Rodrigues L.M.C.S. (2016). Symmetric deformed binomial distributions: An analytical example where the Boltzmann-Gibbs entropy is not extensive. J. Math. Phys..

[218] Amari S., Ohara A. (2011). Geometry of *q*-exponential family of probability distributions. Entropy.

[219] Amari S., Ohara A., Matsuzoe H. (2012). Geometry of deformed exponential families: Invariant, dually-flat and conformal geometries. Phys. A.

[220] Ruiz G., Tsallis C. (2012). Towards a large deviation theory for strongly correlated systems. Phys. Lett. A.

[221] Touchette H. (2013). Comment on “Towards a large deviation theory for strongly correlated systems”. Phys. Lett. A.

[222] Ruiz G., Tsallis C. (2013). Reply to Comment on “Towards a large deviation theory for strongly correlated systems”. Phys. Lett. A.

[223] Tsallis C., Cirto L.J.L. (2014). Thermodynamics is more powerful than the role to it reserved by Boltzmann-Gibbs statistical mechanics. Eur. Phys. J. Spec. Top..

[224] Tsallis C. (2017). Generalization of the possible algebraic basis of *q*-triplets. Eur. Phys. J. Spec. Top..

[225] Alcaraz F.C. (1987). The critical behaviour of self-dual *Z*(*N*) spin systems: Finite-size scaling and conformal invariance. J. Phys. A.

[226] Alcaraz F.C., Martins M.J. (1990). The operator content of the exactly integrable *SU*(*N*) magnets. J. Phys. A.

[227] Tsallis C., Haubold H.J. (2015). Boltzmann-Gibbs entropy is sufficient but not necessary for the likelihood factorization required by Einstein. Europhys. Lett..

[B228-entropy-21-00696] 228.Einstein, A., Ann. Phys. (Leipzig), 33 (1910) 1275: Dass die zwischen S und W in Gleichung (1) [S = R lg W + N konst.] gegebene Beziehung die einzig mogliche ist, kann bekanntlich aus dem Satze abgeleitet werden, dass die Entropie eines aus Teilsystemen bestehenden Gesamtsystems gleich ist der Summe der Entropien der Teilsysteme. [The relation between S and W given in Equation (1) is the only reasonable given the proposition that the entropy of a system consisting of subsystems is equal to the sum of entropies of the subsystems. (free translation by Tobias Micklitz.)]

[B229-entropy-21-00696] 229.Einstein, A., in P.A. Schilpp, Ed. *Autobiographical Notes. A Centennial Edition*. Open Court Publishing Company. 1979. p. 31: A theory is the more impressive the greater the simplicity of its premises is, the more different kinds of things it relates, and the more extended is its area of applicability. Therefore the deep impression that classical thermodynamics made upon me. It is the only physical theory of universal content concerning which I am convinced that, within the framework of applicability of its basic concepts, it will never be overthrown. 1949.

[230] Frigg R., Werndl C. (2019). Can somebody please say what Gibbsian statistical mechanics says?. Br. J. Philos. Sci..

